# Ultralong Carrier Lifetime in Lead-Free Perovskites Enabled by Elimination of Electron–Phonon Coupling in Target Layers

**DOI:** 10.1021/acs.jpclett.6c01910

**Published:** 2026-07-10

**Authors:** Yisen Yao, Liang Pan, Alexander O. Govorov, Arup Neogi, Weiwu Chen, Liujiang Zhou, Zhiming Wang

**Affiliations:** 1 Institute of Fundamental and Frontier Sciences, 12599 University of Electronic Science and Technology of China, Chengdu 610054, China; 2 School of Physics, 12599 University of Electronic Science and Technology of China, Chengdu 611731, P. R. China; 3 Department of Physics and Astronomy, 1354 Ohio University, Athens, Ohio 45701, United States; 4 Shimmer Center, Tianfu Jiangxi Laboratory, Chengdu 641419, P. R. China; 5 Yangtze Delta Region Institute, Huzhou, 12599 University of Electronic Science and Technology of China, Huzhou 313001, P. R. China

## Abstract

Nonradiative electron–hole recombination driven by nonadiabatic coupling (NAC) between band-edge states is the primary bottleneck limiting carrier lifetime and thus the power conversion efficiency (PCE) of photovoltaic and photocatalytic materials. In this work, we demonstrate that NAC in lead-free Ruddlesden–Popper (RP) perovskites can be substantially reduced by identifying and suppressing the electron–phonon (e–ph) coupling in the specific atomic layer that contributes most strongly to carrier recombination, which we term the target layer. Taking Y_2_Ti_2_O_5_S_2_ as a model system, we show that its rock-salt [Y_2_S_2_]^2+^ layer is the dominant source of e–ph coupling. By tuning the spatial distribution of the band-edge states to eliminate their overlap within this target layer, the NAC of Ti-based (and Zr-based) RP perovskites is reduced to 0.18 meV, comparable to that of lead halide perovskites (0.16 meV). Time-dependent density functional theory (TDDFT) simulations yield a carrier lifetime of ∼0.8 μs for the designed material Ba_3_Zr_2_O_5_S_2_ at room temperature, surpassing that of c-CsPbI_3_ (∼0.4 μs). This strategy of controlling NAC through target-layer engineering provides new insight into carrier dynamics and offers a practical guideline for designing lead-free perovskites with improved PCE.

## Graphical abstract

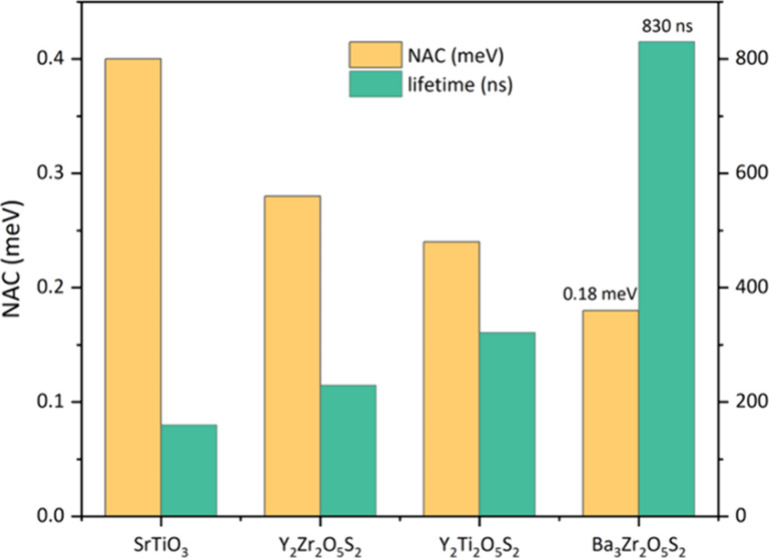

Carrier lifetime can significantly influence energy conversion efficiency in many fields, such as photovoltaics and photocatalysis (Figure 1a).^1–11^ It is mainly determined by the nonadiabatic electron–phonon coupling (NAC) between the conduction band minimum (CBM) and the valence band maximum (VBM). Conventional methods to reduce the NAC for a long carrier lifetime in lead halide perovskites include temperature effects, built-in electric field, large polaron, and edge effects,^12–16^ which mainly focus on the separation of electron–hole (e–h) to decrease the CBM-VBM’s electron–phonon coupling. However, these approaches result in a shorter carrier lifetime and limit improvements in the power conversion efficiency (PCE) in lead-free perovskites due to the strong electron–phonon coupling, as observed in Ti-based perovskites^17–23^ (Table 1S). It necessitates a disruptive design strategy at the atomic level to explore novel lead-free perovskites with decreased NAC, ultralong carrier lifetime, and high PCE.

Y_2_Ti_2_O_5_S_2_, a cation-deficient Ruddlesden–Popper (RP) perovskite, which has been prepared and exhibits wide applications in many fields for decades,^24–29^ has attracted more and more attention due to its stability and relatively high efficiency.^30–33^ In these years, K. Domen et al. found that Y_2_Ti_2_O_5_S_2_ could be a photocatalyst for overall water splitting without sacrificial agents.^30^ The relatively high apparent quantum yield (AQY) efficiency (5%) has been experimentally attributed to the ultralow recombination rate of free carriers and long carrier lifetime.^32,33^ While investigating these remarkable properties’ underlying physical reasons, we discovered varying contributions of different layers to the electron–phonon (e–ph) coupling, which may enable us to achieve extended carrier lifetime by eliminating the e–ph coupling from the layer with the highest contribution. However, this hypothesis needs to be further investigated systematically via ab initio nonadiabatic molecular dynamics (NAMD) to verify the design concept of eliminating the e–ph coupling of the target layer.

Here, we demonstrate that eliminating the e–ph coupling of the target layer (the layer that contributes most strongly to NAC) in cation-deficient RP perovskites reduces NAC to an ultralow value and yields a long carrier lifetime. We first quantify the unequal contributions of different atomic layers to e–ph coupling in Y_2_Ti_2_O_5_S_2_. By tuning the spatial distribution of the band-edge states, we then eliminate their overlap within the rock-salt [Y_2_S_2_]^2+^ layer the target layer, thereby suppressing the dominant e–ph coupling pathway and achieving ultralow NAC with an ultralong carrier lifetime in lead-free RP perovskites (Figure 1). Through decomposition of the vibrational modes involved in e–ph coupling and analysis of band-edge wave function overlap, we identify the primary contributors to NAC. The NAC of the designed material Ba_3_Zr_2_O_5_S_2_ is reduced to 0.18 meV, on par with that of c-CsPbI_3_ (0.16 meV), yielding a microsecond-scale carrier lifetime of ∼0.8 μs.

**Figure 1:**
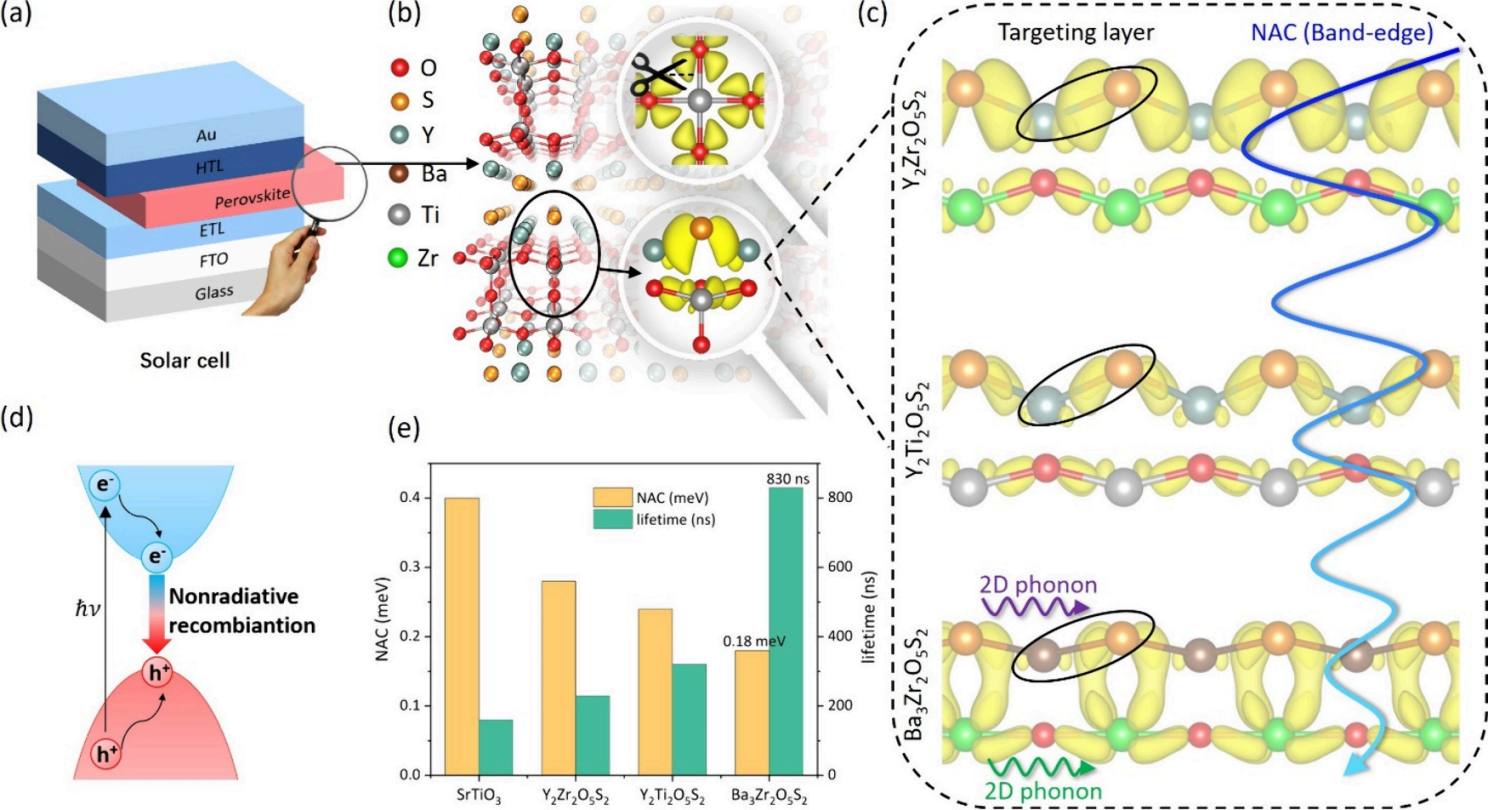
Extending the carrier lifetime by reducing NAC in the target layer. (a) Schematic of the perovskite solar cell (PSC) structure and the materials investigated in this study. (b) Comparison of e–ph coupling characteristics between SrTiO_3_ and Y_2_Ti_2_O_5_S_2_. The yellow regions represent the spatial overlap between CBM and VBM wave functions. The breaking of the Ti–S covalent bond eliminates the overlap between Ti and S atoms; however, overlaps within the Y–S and Ti–O layers remain. (c) Separation of the rock-salt layer and perovskite layer in Y_2_Zr_2_O_5_S_2_ (top), Y_2_Ti_2_O_5_S_2_ (middle), and Ba_3_Zr_2_O_5_S_2_ (bottom). The yellow regions indicate the change in spatial overlap between CBM and VBM, which shifts from intralayer (Y_2_Zr_2_O_5_S_2_) to interlayer (Ba_3_Zr_2_O_5_S_2_). The atoms enclosed by the black ellipse constitute the target layer, the layer whose e–ph coupling is selectively eliminated in our design strategy. (d) Schematic of nonradiative electron–hole recombination investigated in this work. The blue region represents the conduction band; the red region represents the valence band. (e) As the overlap of band-edge states within the target layer is progressively reduced, the NAC decreases substantially and the carrier lifetime increases correspondingly.

TiO_6_ octahedrons’ symmetry breaking induced decoupling. The symmetry group of Y_2_Ti_2_O_5_S_2_ is *I*4/*mmm*, and its calculated lattice constants (*a* = *b* = 3.79 Å, *c* = 23.0 Å) are identical to the data in experiments.^34^ Y_2_Ti_2_O_5_S_2_ can be seen as an intergrowth structure of rock-salt slabs [Y_2_S_2_]^2+^ and –(TiO_2_)–(O)–(TiO_2_)– layers (simplified as [Ti_2_O_5_]^2–^ layer) stacked along the *z*-direction (Figure 1b). It can also be seen as the vertex O atom of the TiO_6_ octahedron is replaced by an S atom; hence the Ti–O covalent bond is replaced by Ti–S ionic bond (Figure 1b). The far distance (2.9 Å) and the low electron localization function (ELF) between Ti and S atoms indicate Ti–S bond is an extremely weak chemical bond, which supports the atomic vibration decoupling between Ti and S. Notably, Ti and O atoms form covalent bonds and spread all over the *xy* plane continuously, which provide a transport probability for electrons.^35–38^ Y_2_Ti_2_O_5_S_2_ has a direct bandgap. Its CBM is mainly contributed by the d_*xy*_ orbital of Ti atoms, and the VBM is mainly contributed by the p_*x*_ or p_*y*_ orbital of S atoms. However, there is no overlap between Ti d_*xy*_ and S p_*x, y*_ orbitals, which indicates no contribution to band-edge states’ NAC.^35,36^ In comparison, the band-edge states overlap is mainly contributed by Y and S atoms (Figure 1S), which is the main reason that cause the e–h recombination in Y_2_Ti_2_O_5_S_2_, as shown in Figure 1c. It explains the low NAC in Y_2_Ti_2_O_5_S_2_ and the long carrier lifetime of Y_2_Ti_2_O_5_S_2_ observed in experiments (Figure 1d,e).^33^ Furthermore, the vibration modes of atomic pairs well explains^39,40^ the superhigh free-carrier concentration rarely observed in experiments.^32^

In the study of e–ph coupling in dynamics, to further confirm the influence of e–ph coupling on the e–h recombination process in Y_2_Ti_2_O_5_S_2_, Y_2_Ti_2_O_5_S_2_’s ab initio molecular dynamics (AIMD) were studied. SrTiO_3_ is the reference material for comparison due to its close bandgap proximity with Y_2_Ti_2_O_5_S_2_ and similar atomic masses of Sr and Y atoms.^41^ The high stability and its environmental-friendly properties have attracted increased research activities on SrTiO_3_.^38,42–44^ The dynamic population of band-edge states in SrTiO_3_ is in Figure 2a. In SrTiO_3_, CBM is mainly contributed by Ti atoms, and VBM is mainly contributed by O atoms. Ti and O atoms form the covalent bond, exhibiting a strong vibrational coupling.^37,38^ Hence, the NAC is high, as shown in Table 1, and the coupled vibrational oscillation between Ti and O atoms may lead to massive electron–hole (e–h) recombination. As shown in Figure 2a, VBM and CBM’s dynamic distributions strongly correlate with atomic vibration, which coincides with its high NAC. These provide strong validation for SrTiO_3_’s short carrier life than lead halide perovskite because of well-defined normal modes causing electron dispersed distribution observed in experiments.^45^ For Y_2_Ti_2_O_5_S_2_, the breakdown of the symmetry of TiO_6_ octahedrons leads to the atomic vibration decoupling between Ti and S atoms. Furthermore, it leads to decreased e–ph coupling with significantly reduced NAC (Figure 1e). Figure 2b shows that band-edge distributions remain relatively stable, and the temporal asynchronous behavior is evident from the changes in CBM and VBM over time. The orbital overlap near the Fermi-level mainly happened between Ti atoms and equator O atoms and between Y atoms and S atoms^35,36^ (Figure 1c). Hence, the band-edge’s NAC is mainly contributed by the e–ph coupling around Y–S or Ti–O bonds. In our previous work,^35,36^ we utilized the normal-mode-decomposition technique^46,47^ to obtain the phonon density of states (DOSph) at 300 K and visualize the vibrational modes participating in carrier recombination. It was confirmed that the vibrational coupling between Ti and S atoms is weak due to their large frequency differences. Most importantly, our previous study has shown that the phonon mode that participates in gap changing mainly occurs at 283.5 cm^–1^ and 567.3 cm^–1^ position,^35,36^ which can be assigned to the atomic vibration modes from Y–S bonds within the rock-salt layer and the Ti–O bonds within the perovskite layer, respectively. These peaks also align well with the overlapping region of Y_2_Ti_2_O_5_S_2_, as shown in Figure 1c. The highest peak indicates that the Y–S bond vibration mainly contributes to the gap changes, thereby also serving as the main contributor to e–ph coupling.^35,36^ As Y–S bond vibration (in the rock-salt layer) greatly influences NAC, the elimination of e–ph coupling in the rock-salt layer for the reduction of the NAC is the main topic that has been undertaken.

**Figure 2:**
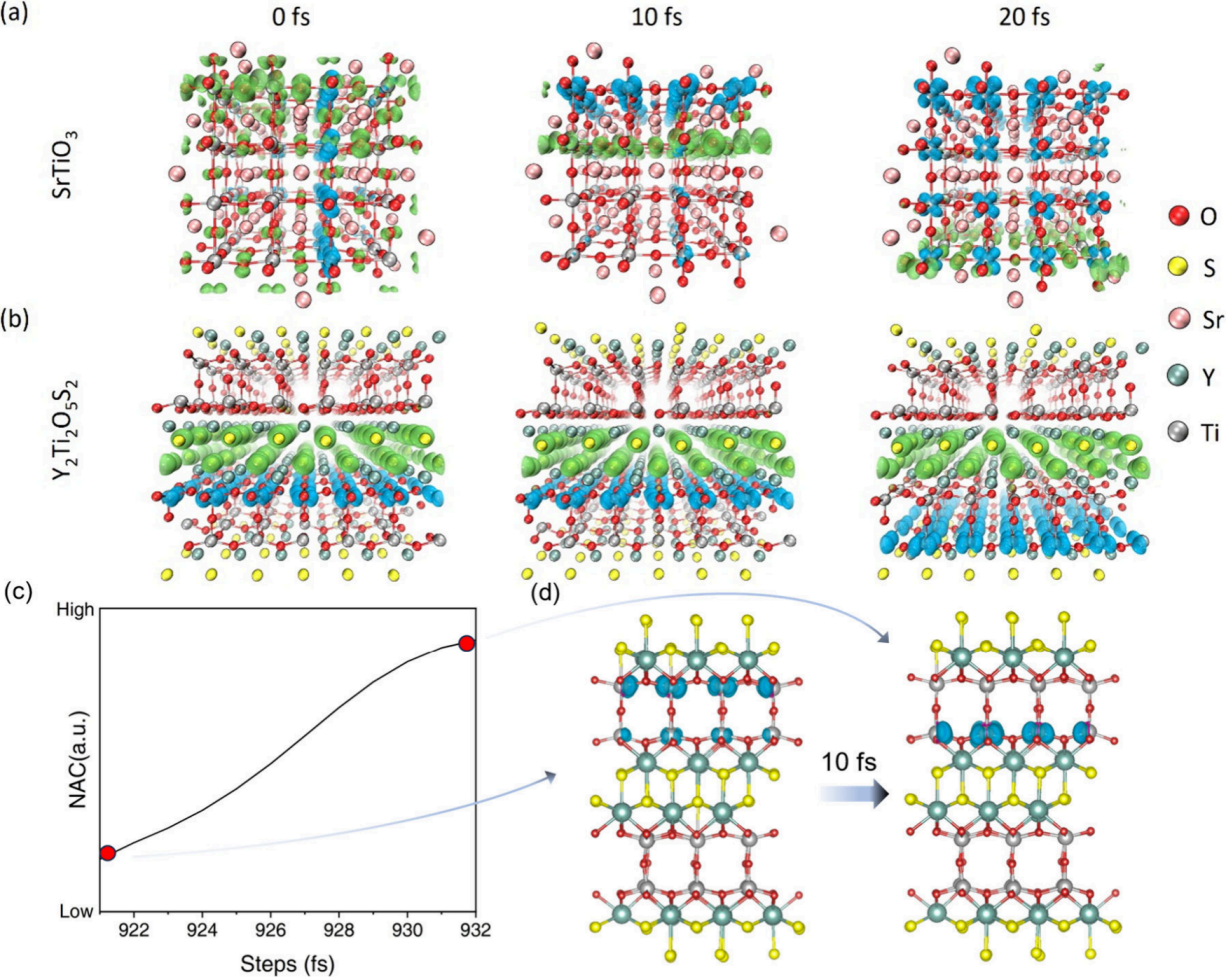
Dynamic characterization of Y_2_Ti_2_O_5_S_2_. (a, b) VBM (green) and CBM (blue) distributions of SrTiO_3_ (a) vs Y_2_Ti_2_O_5_S_2_ (b) for the snapshots at 0 fs (left), 10 fs (middle), and 20 fs (right) in dynamics at 300 K. The distribution of VBM and CBM in SrTiO_3_ exhibits significant temporal variation, while their distribution in Y_2_Ti_2_O_5_S_2_ is relatively stable over time. (c) Selected time interval during the AIMD trajectory in which the NAC of Y_2_Ti_2_O_5_S_2_ evolves from low to high values. We selected the NAC values within the range of 921 to 932 fs and took the CBM distributions at the minimum value (at 921 fs) and maximum value (at 932 fs) for further observation and analysis. (d) Spatial distribution of the CBM of Y_2_Ti_2_O_5_S_2_ at specific time points during the AIMD trajectory, corresponding to the time points marked in (c). It can be observed that as the density distribution changes from the upper portion to the lower portion, the NAC in (c) increases from low to high. The detailed evolution at each fs is provided in Figure 2S.

**Table 1:** Bandgap (*E*_g_), Nonadiabatic Electron–Phonon Coupling (NAC), Pure-Dephasing Time (τ_2_), Standard Deviation of Bandgap Fluctuation (σ_E_), Carrier Lifetimes (τ_1_), and the Atomic Number (*N*) We Used in Simulation for Charge Recombination in CsPbX_3_ (X = Cl, Br, I), SrTiO_3_, Y_2_Ti_2_O_5_S_2_, Y_2_Zr_2_O_5_S_2_, and Ba_3_Zr_2_O_5_S_2_

	*E*_g_ (eV)	NAC (meV)	τ_2_ (fs)	σ_E_ (meV)	τ_1_ (ns)	*N*
CsPbCl_3_	2.90	0.60	5.0	63.8	39	40
CsPbBr_3_	2.67	0.37	5.9	78.8	165	40
CsPbI_3_	2.00	0.16	6.4	67.0	479	40
SrTiO_3_	1.84	0.40	15.4	37.4	160	320
Y_2_Ti_2_O_5_S_2_	1.84^a^	0.24	8.1	58.4	321	198
Y_2_Zr_2_O_5_S_2_	2.10	0.28	7.0	51.8	229	198
Ba_3_Zr_2_O_5_S_2_	2.02	0.18	9.5	57.3	830	198

a Obtained from experimental data.^31^

Tuning the band-edge states’ distribution decreases the rock-salt layer’s overlap. We designed Y_2_Zr_2_O_5_S_2_ and Ba_3_Zr_2_O_5_S_2_ (Table 2S and Figure 3S) to explore further the influences of band-edge states’ distribution around the Y–S bond on NAC and carrier lifetime (Figure 3a and Figure 2c,d). Y_2_Zr_2_O_5_S_2_ and Ba_3_Zr_2_O_5_S_2_’s structure is presented in Figure 4S. We can find that they both have a similar crystal structure as Y_2_Ti_2_O_5_S_2_. It is evident from Figure 5S and Figure 6S that they exhibit similar phonon spectra, indicating that their vibrational modes also remain comparable. This is a crucial prerequisite for our ability to regulate e–ph coupling by modifying the distribution of band-edge states. To investigate the influence of the wave function’s distribution on NAC, we plotted the changing of the spatial distributions of band-edge states’ wave functions as NAC increases during the molecular dynamics process (Figures 7S–9S). We observed that, in the molecular dynamics process of any given material, as NAC increased, the spatial distributions of VBM and CBM wave functions became increasingly adjacent. This indicates that the overlap of band-edge states plays a decisive role in NAC. Moreover, as shown in Figure 2c,d, the contribution of CBM to NAC is significant, indicating that tuning the CBM distribution provides an effective means of modulating NAC. For Y_2_Ti_2_O_5_S_2_, the S 3p_*x,y*_ orbital’s energy is higher than O 2p_*x,y*_’s. Hence the VBM is mainly contributed by the S atom. As shown in Figure 1c, we find that the CBM-VBM overlap mainly happened around the Y–S bond. From the partial density of states (PDOS), we can also find that the Y atom mainly contributes to the VBM and CBM (Figure 3g). For Y_2_Zr_2_O_5_S_2_, the Zr 3d orbital’s energy is higher than Ti 4d orbital’s, and the electronegativity of the Zr atom is much closer to the Y atom. Hence, the CBM is more likely to distribute around the Y atom than Y_2_Ti_2_O_5_S_2_. As seen in Figure 3b, we can find that Y atoms contribute more to CBM in Y_2_Zr_2_O_5_S_2_ compared with that in Y_2_Ti_2_O_5_S_2_ (Figure 3b left). The PDOS of Y_2_Zr_2_O_5_S_2_ also showed that the contribution of the Y atom increased compared with that in Y_2_Ti_2_O_5_S_2_ (Figure 3f). In both structures, the Y atoms are coordinated by four S atoms, and the cation-deficient position are coordinated by four O atoms, which allows removing the CBM distribution around the Y atom through filling atoms into cation-deficient position. For Ba_3_Zr_2_O_5_S_2_, the d_*xy*_ orbital of Ba atoms in the rock-salt layer coordinated with S atoms has higher energy than that of Ba atoms coordinated with O atoms because of S atoms’ bigger radius. Hence, the Ba atoms coordinated with O atoms mainly contribute to the CBM, while Ba atoms in the rock-salt layer mainly contribute to the VBM (Figure 3h). The overlap in the rock-salt layer disappeared, and the overlap of CBM-VBM mainly occurs between Zr and S atoms (Figure 1c). The atomic vibration between Zr and S atoms exhibits a weak correlation, ensuring the overlap between Zr and S atoms has little contribution to the e–ph coupling in NAC.

**Figure 3:**
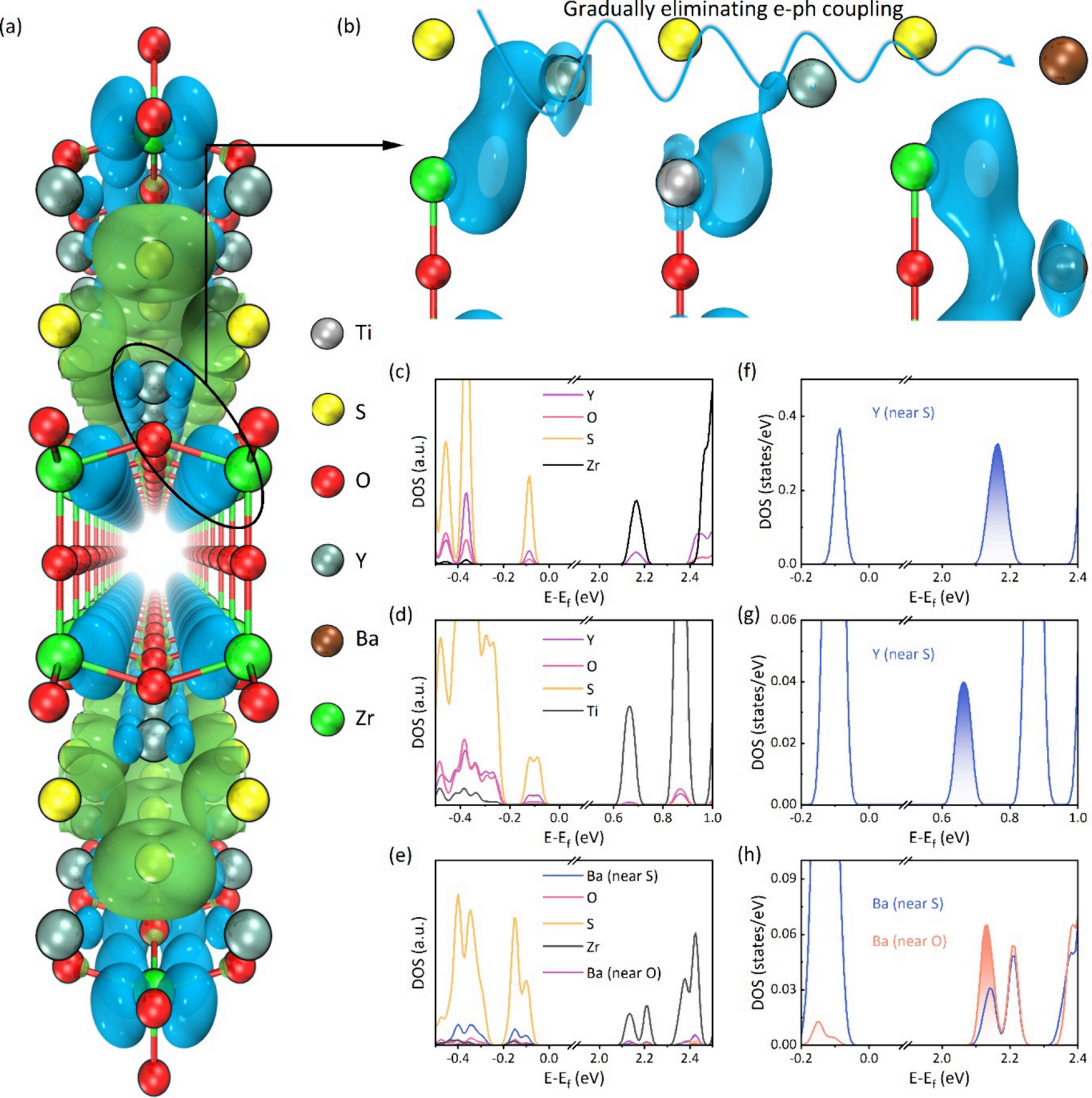
Adjustment of CBM states’ distribution. (a) The static distribution of VBM (green) and CBM (blue) in Y_2_Zr_2_O_5_S_2_. The isosurface value is 0.001 e/Å^3^. Though the VBM and CBM are principally segregated in different layers, there exists a minor overlap of band-edge states within the [Y_2_S_2_]^2–^ layer. (b) The zoom-in view of CBM’s distribution in Y_2_Zr_2_O_5_S_2_ (left), Y_2_Ti_2_O_5_S_2_ (middle), and Ba_3_Zr_2_O_5_S_2_ (right) with isosurface value 0.0005 e/Å^3^. We can find that the CBM’s distribution around cations in the rock-salt layer disappears in Ba_3_Zr_2_O_5_S_2_. (c–e) The DOS in Y_2_Zr_2_O_5_S_2_ (c), Y_2_Ti_2_O_5_S_2_ (d), and Ba_3_Zr_2_O_5_S_2_ (e). (f–h) The PDOS of the CBM of Y_2_Zr_2_O_5_S_2_ (f), Y_2_Ti_2_O_5_S_2_ (g), and Ba_3_Zr_2_O_5_S_2_ (h). We mainly draw the cation’s contribution to the band-edge to show the result of the band-edge adjustment. The shadow of the peak position shows the second contribution to CBM as the second contributor mainly influences the NAC in our case.

For the effects of band-edge state adjustment on e–ph coupling, the dynamics of the excited states are investigated and presented in Figure 4 based on time-dependent density functional theory (TDDFT). Figure 4b provides the dynamic band-edge states overlaps in SrTiO_3_, Y_2_Zr_2_O_5_S_2_, Y_2_Ti_2_O_5_S_2_, and Ba_3_Zr_2_O_5_S_2_. The overlap between CBM and VBM changes dynamically in molecular dynamics, mainly determined by the bonding character. It is observed that the static overlap in Y_2_Zr_2_O_5_S_2_ is higher than that in Y_2_Ti_2_O_5_S_2_ (Figure 4a). They both have two significant peaks in dynamic overlap, and Y_2_Zr_2_O_5_S_2_’s peaks are higher than Y_2_Ti_2_O_5_S_2_’s, which coincides with the increased NAC in Y_2_Zr_2_O_5_S_2_ and provides further evidence for the predominant contribution of the overlap around the Y–S bond to NAC. For Ba_3_Zr_2_O_5_S_2_, the spatial distribution of the dynamic overlap is more delocalized than others (Figure 4b) owing to the weak vibrational coupling between rock-salt layer and perovskite layer as shown in Figure 1c. Hence the NAC of Ba_3_Zr_2_O_5_S_2_ is much smaller than that of Y_2_Zr_2_O_5_S_2_. The Fourier transforms (FTs) of the NAC values’ autocorrelation functions are named as influenced spectral densities and can also be demonstrated in Figure 10S. It is observed that the phonon modes that participate in NAC in Ba_3_Zr_2_O_5_S_2_ is much fewer than that in Y_2_Zr_2_O_5_S_2_. It further confirms the obvious effect of the removal of the e–ph coupling in the target layers on NAC. The Ba_3_Zr_2_O_5_S_2_’s NAC even reaches the same level as c-CsPbI_3_’s. In lead-based perovskites, the ultralow NAC is always an important character that guarantees c-CsPbI_3_ as one of the most promising materials in the area of photovoltaics (Table 1).^48–50^ The average NAC in a period (such as 2 ps in our cases) cannot thoroughly describe the e–ph interaction observed in experiments,^50–54^ so we visualized the NAC numerical distribution through violin plot using kernel smoother technique (Figure 4c). We find that the fluctuations of NAC decreased significantly as the e–ph coupling of the rock-salt layer is gradually attenuated. It is similar to the characteristic of NAC in lead-halide perovskites where the NAC is reduced with an increase in the element’s mass (Figure 4c left).^55–61^ This manipulation of eliminating the e–ph coupling of the target layer dramatically influences NAC, providing a new pathway to find alternatives to lead-based perovskites. We also find that there should be more than one polaron in lead-halide perovskite because of the several nodes shown in the NAC violin plot (Figure 4c). This is in good agreement with the multipolaron phenomenon previously reported by other research groups.^53,54,62^ As observed in the violin plot, there is only one broad node near the zero line in Ti-based (or Zr-based) perovskites compared to the several nodes in lead-based perovskite. It further strengthens the robustness of band-edge states’ separated distribution and coincides with the harmonicity of thermal fluctuations as experimentally observed in Ti-based perovskite.^45^ The lifetime simulated in Figure 4d coincides with the NAC fluctuation trend list in Figure 4c. It is worth mentioning that the Ba_3_Zr_2_O_5_S_2_’s lifetime is even much longer than that of CsPbI_3_ (Table 1). Most importantly, the material Ba_3_Zr_2_O_5_S_2_ is stable because Y_2_Ti_2_O_5_S_2_’s stability has been demonstrated in experiments, and they both have a significant hybridization between S and O atoms, in which S^2–^ is stable (Figures 5S and 11S).

**Figure 4:**
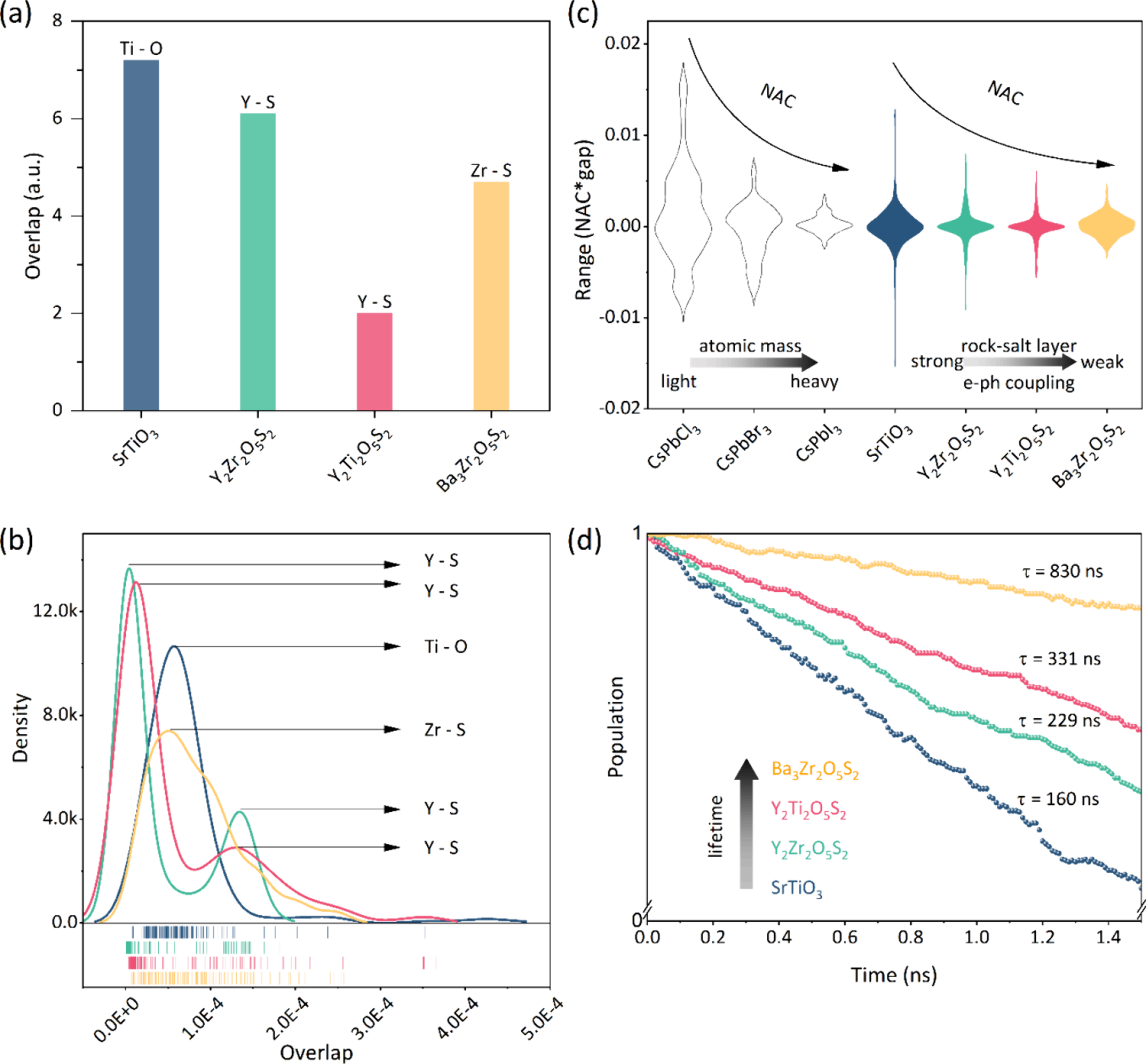
Influence of band-edge states adjustment on e–h recombination. (a) The static overlaps of VBM-CBM partial states in SrTiO_3_, Y_2_Zr_2_O_5_S_2_, Y_2_Ti_2_O_5_S_2_, and Ba_3_Zr_2_O_5_S_2_. (b) The dynamic overlaps of VBM-CBM partial states in SrTiO_3_, Y_2_Zr_2_O_5_S_2_, Y_2_Ti_2_O_5_S_2_, and Ba_3_Zr_2_O_5_S_2_ at 300 K. The data are processed using kernel smoother technique. The rug plot (lower) is plotted to visualize the data distribution. (c) The violin plot (kernel smoother technique) of NAC fluctuation for each material obtained from molecular dynamics. We can find that our design method has the same effect with lead halide perovskites. (d) Electron population decay in SrTiO_3_ (blue), Y_2_Ti_2_O_5_S_2_ (cyan), Y_2_Zr_2_O_5_S_2_ (orange), and Ba_3_Zr_2_O_5_S_2_ (red) at room temperature. Due to its much weaker NAC, the simulated electron–hole recombination time in Ba_3_Zr_2_O_5_S_2_ is much longer than that in Y_2_Ti_2_O_5_S_2_.

For potential application of Ba_3_Zr_2_O_5_S_2_ in solar cells, it is worth mentioning that Ba_3_Zr_2_O_5_S_2_ is poised to become a highly promising alternative to lead halide perovskites in the field of solar cells. The remarkable progress of hybrid perovskite solar cells over the past decade can be primarily attributed to their strong polarization effect, leading to an exceptionally low e–ph coupling and excellent carrier-related properties.^48–50^ In contrast, Ba_3_Zr_2_O_5_S_2_ achieves a significantly reduced e–ph coupling through modulation, setting it apart from other lead-free perovskites.^45^ Its analogue compound, Y_2_Ti_2_O_5_S_2_, has been experimentally characterized to exhibit the same carrier recombination rate as hybrid perovskites, as well as high stability for overall water splitting.^30^ With a tunable bandgap similar to that of hybrid perovskites, the materials of this family possess a direct bandgap that is highly favorable for light absorption. Moreover, it achieves compositional nontoxicity. Furthermore, this type of structure may also possess a more compatible lattice with electron transport layer (ETL) materials,^4^ in solar cells, such as TiO_2_, promoting effective separation of electrons and holes. In terms of light absorption, it can achieve ultrastrong light absorption like lead halide perovskites through material design. In fact, based on such materials, we have also designed materials that combine ultrastrong light absorption and ultralow e–ph coupling, which will be published as our next work. It is worth mentioning that the suppressed e–ph coupling within the target layer discovered in this work can be directly probed using time-resolved spectroscopic techniques. For instance, transient absorption spectroscopy (TAS) can resolve carrier recombination dynamics with femtosecond resolution, allowing the carrier lifetime to be obtained.^23^

In this work, we attribute the long carrier lifetime of Y_2_Ti_2_O_5_S_2_ to the low NAC resulting from vibrational decoupling between Ti and S atoms. Through combined analysis of static electronic structure and dynamic carrier properties, we identify the rock-salt [Y_2_S_2_]^2+^ layer as the primary contributor to the overall e–ph coupling in Y_2_Ti_2_O_5_S_2_ and therefore as the target layer for e–ph coupling elimination. Building on this insight, we demonstrate that the e–ph coupling in the rock-salt layer can be maximally suppressed by tuning the spatial distribution of the CBM states, which dramatically reduces the NAC. Our results show that controlling the spatial distribution of band-edge states, which is equivalent to controlling the e–ph coupling in these systems, has a decisive impact on both NAC and carrier lifetime. Using only light, earth-abundant elements, we achieve ultralow NAC and ultralong carrier lifetime in lead-free RP perovskites, challenging the conventional paradigm that high-performance perovskites must contain heavy elements such as Pb. Additionally, our analysis of NAC fluctuations suggests that multiple polaron species may coexist in lead halide perovskites. Our findings establish a general strategy (eliminating the e–ph coupling in a specific target layer) for extending carrier lifetime, opening new avenues for the discovery and design of efficient lead-free photovoltaic and photocatalytic materials.

## Computational Methods

All the calculations were performed based on the density functional theory (DFT) and plane-wave basis, as implemented in Vienna Ab Initio Simulation Package (VASP).^53–65^ The exchange–correlation function was treated by the Perdew–Burke–Emzerhof (PBE) parametrized gradient approximation (GGA). A plane wave basis of 520 eV was employed. In the optimization process, all the atoms in the unit cell were fully relaxed until the force per atom was smaller than 0.02 eV/Å, and the lattice was fixed. The PBE functional calculation was mainly used in this work as it presents a similar electronic structure as HSE06 ^66^ except that the bandgap will not affect the main problem discussed in the preceding section. The spin–orbital coupling (SOC) was found to have an insignificant effect on the band structure and has not been considered in the calculation process. The Brillouin zone was sampled by an 11 × 11 × 3 k-point mesh to calculate the primitive cell. Crystal orbital Hamilton populations (COHP) were calculated using Lobster.^67^ Based on the mix quantum-classical calculation (MQC),^68–71^ Hefei-NAMD packages were used to perform the semiclassical decoherence-induced surface hopping (DISH) calculation within the framework of TDDFT.^71^ The computation of NAMD trajectories and Kohn–Sham orbitals was executed based on the density functional theory and plane-wave basis, as implemented in VASP. Molecular dynamics simulations (MD) were carried out at the Γ-point with 3 × 3 × 1 for all the *I*4/*mmm* group crystals. A 5 ps Born–Oppenheimer MD simulation was carried out using repeated velocity rescaling to bring the temperature from 0 to 300 K until the equilibrium temperature was reached. Subsequently, 5000 fs microcanonical Born–Oppenheimer MD trajectories were obtained with 1 fs time step and were utilized for the subsequent NAC calculations.

## Supplementary Material



## Notes

Band structures of Y_2_Ti_2_O_5_S_2_, Y_2_Zr_2_O_5_S_2_, Ba_3_Zr_2_O_5_S_2_; electron wave function’s evolution; phonon spectra of materials; the formula we used in this paper; lead-free perovskites’ efficiency in solar cell; the lattice parameters we calculated; calculated effective mass of *m*_h_ and *m*_e_; vibration modes that participate in NAC; the energy changing in AIMD simulation; exciton wave function distribution (PDF)
