# Supplementary material for: Ultralong Carrier Lifetime in Lead-Free Perovskites Enabled by Elimination of Electron–Phonon Coupling in Target Layers
Source: J Phys Chem Lett. 2026 Jul 10;17(29):8309–17. doi: 10.1021/acs.jpclett.6c01910 (PMC13403310; doi:10.1021/acs.jpclett.6c01910)
Supplement: Supplementary file 1 [file jz6c01910_si_001.pdf]

Supporting Information for:

Ultralong Carrier Lifetime in Lead-Free Perovskites Enabled by Elimination of Electron-Phonon Coupling in Target Layers

Yisen Yao[1,2], Liang Pan[1], Alexander O. Govorov*[3], Arup Neogi[1,4], Weiwu Chen[5], Liujiang Zhou*[2], Zhiming Wang*[2,4]*

[1]Institute of Fundamental and Frontier Sciences, University of Electronic Science and Technology of China, Chengdu 610054, China.

[2]School of Physics, University of Electronic Science and Technology of China, Chengdu 611731, P. R. China.

[3]Department of Physics and Astronomy, Ohio University, Athens, Ohio 45701, United States.

[4]Shimmer Center, Tianfu Jiangxi Laboratory, Chengdu, 641419 P. R. China

[5]Yangtze Delta Region Institute (Huzhou), University of Electronic Science and Technology of China, Huzhou 313001, P. R. China.

	Device configuration	VOC /V	JSC /(mA*cm^{-2})	FF	PCE /%	Year	Ref
Double perovskites	FTO/c-TiO_2/mp-TiO_2 /$Cs_2AgBiBr_6$/ Spiro-OMeTAD/Au	1.08	3.55	0.65	2.47	2022	1[1]
	FTO/c-TiO_2/mp-TiO_2 /$Cs_2AgBiBr_6$/ Spiro-OMeTAD/Au	1.09	3.2	0.68	2.3	2021	2[2]
	FTO/c-TiO_2/mp-TiO_2 /$Cs_2AgBiBr_6$/Carbon	1.2	3.15	0.69	2.57	2020	3[3]
	FTO/TiO_2/$Cs_2AgBiBr_6$ /Spiro-OMeTAD/Au	0.98	3.93	0.63	2.4	2017	4[4]
Sn-based perovskites	$FASnI_3$/$NaBH_4$ /DipI-Forward	0.66	22.13	0.73	10.61	2022	5[5]
	ITO/PEDOT: PSS/$FASnI_2$/ICBA/BCP/Ag	0.92	21	0.77	14.6	2021	6[6]
	ITO/PEDOT: PSS/$FASnI_2$/ICBA/BCP/Ag	0.94	17.4	0.75	12.4	2020	7[7]
	ITO/PEDOT:PSS/$FASnI_2$/C_{60}/BCP/Ag	0.56	23.34	0.74	9.61	2019	8[8]
Other perovskites	ITO/PEDOT: PSS/ $MAGeI_{2.7}Br_{0.3}$/PC70BM /Ag	0.45	2.8	0.45	0.57	2018	9[9]
	FTO/TiO_2/$(CH_3NH_3)_2CuX$/Spiro-OMeTAD/Au	0.56	8.12	0.53	2.41	2018	10[10]
	FTO/TiO_2/$Cs_3Sb_2I_9$/Au	0.61	3.55	0.56	1.21	2019	11[11]
	ITO/SnO_2/$AgBiI_4$/PTAA/Au	0.83	5.07	0.67	2.5	2019	12[12]
...	…	…	…	…	…	…	…

Table 1S. The summary of typical lead-free perovskite devices performance

We can see from the above table that the efficiency of non-perovskite materials is still very low, and in recent years, it has been difficult to make significant improvements in the efficiency of non-perovskite materials.

	Lattice parameter	α=β=γ	Space group	Number of atoms
$SrTiO_3$	a=b=c=3.95	90	$Pm\bar{3}m$	320
$Y_2Zr_2O_5S_2$	a=b=3.94, c=23.84	90	I4/mmm	198
$Y_2Ti_2O_5S_2$	a=b=3.79, c=23.04	90	I4/mmm	198
$Ba_3Zr_2O_5S_2$	a=b=4.31, c=24.48	90	I4/mmm	198

Table 2S. Calculated lattice parameters. The number of atoms means the numbers of atoms we used in NAMD simulation. The lattice constants of $SrTiO_3$ and $Y_2Ti_2O_5S_2$ are consistent with the experimental data, which provides a reference for the preparation of $Ba_3Zr_2O_5S_2$.

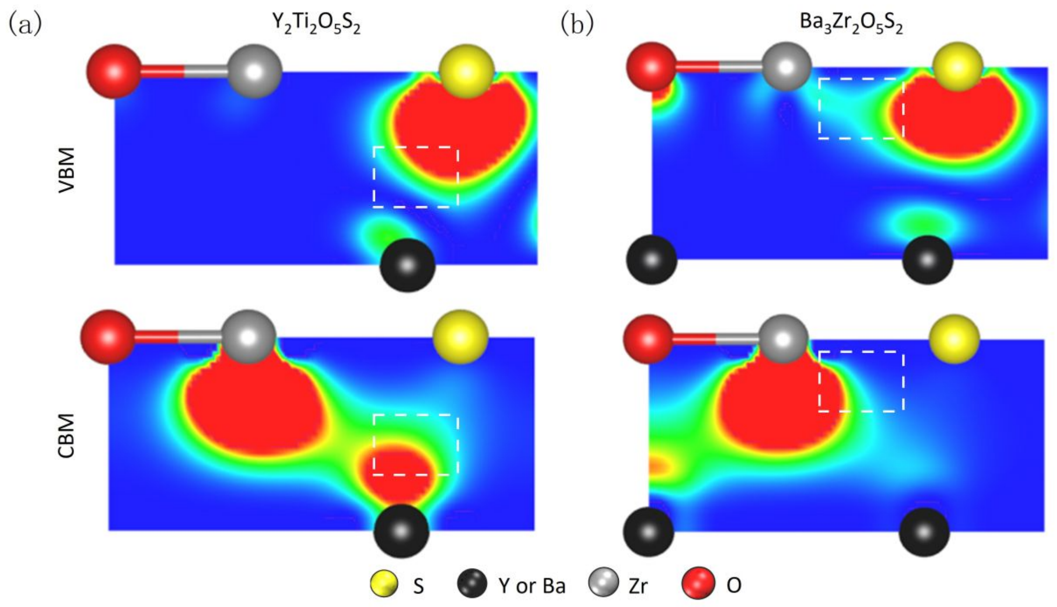

Figure 1S. (a–b) The sectional drawing of VBM (up) and CBM's (down) distributions of $Y_2Ti_2O_5S_2$ (a) vs $Ba_3Zr_2O_5S_2$ (b). The bright areas (red and green) represent the band-edge's mainly distribution area. The white dashed box indicates the region of predominant overlap between CBM and VBM. In $Y_2Ti_2O_5S_2$, the overlap between VBM and CBM primarily takes place in the Y–S region, while in $Ba_3Zr_2O_5S_2$, the overlap mainly occurs in the Zr–S region.

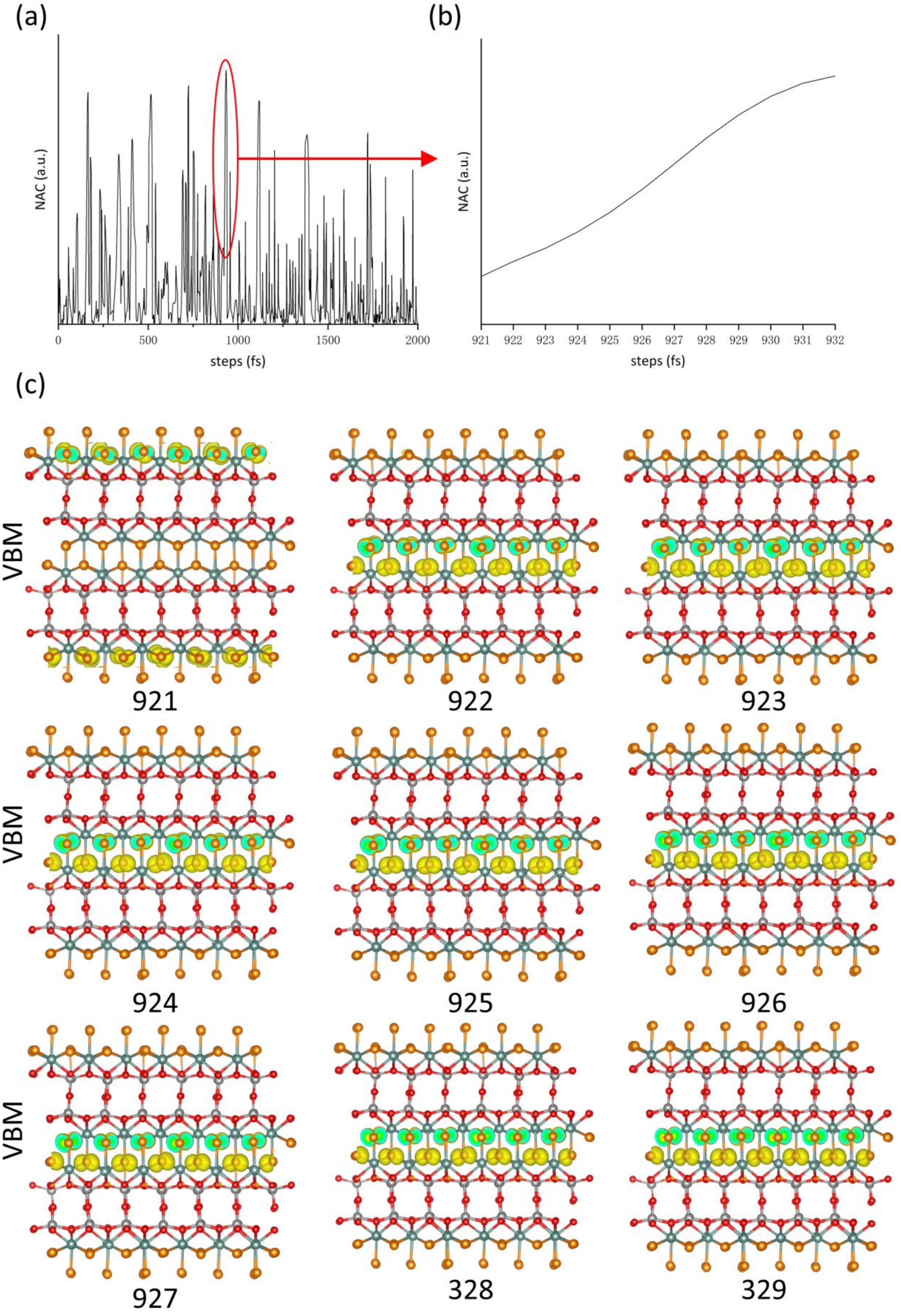
(a)
NAC (a.u.)
steps (fs)
0
500
1000
1500
2000
(b)
NAC (a.u.)
steps (fs)
921
922
923
924
925
926
927
928
929
930
931
932
(c)
VBM
921
922
923
VBM
924
925
926
VBM
927
328
329

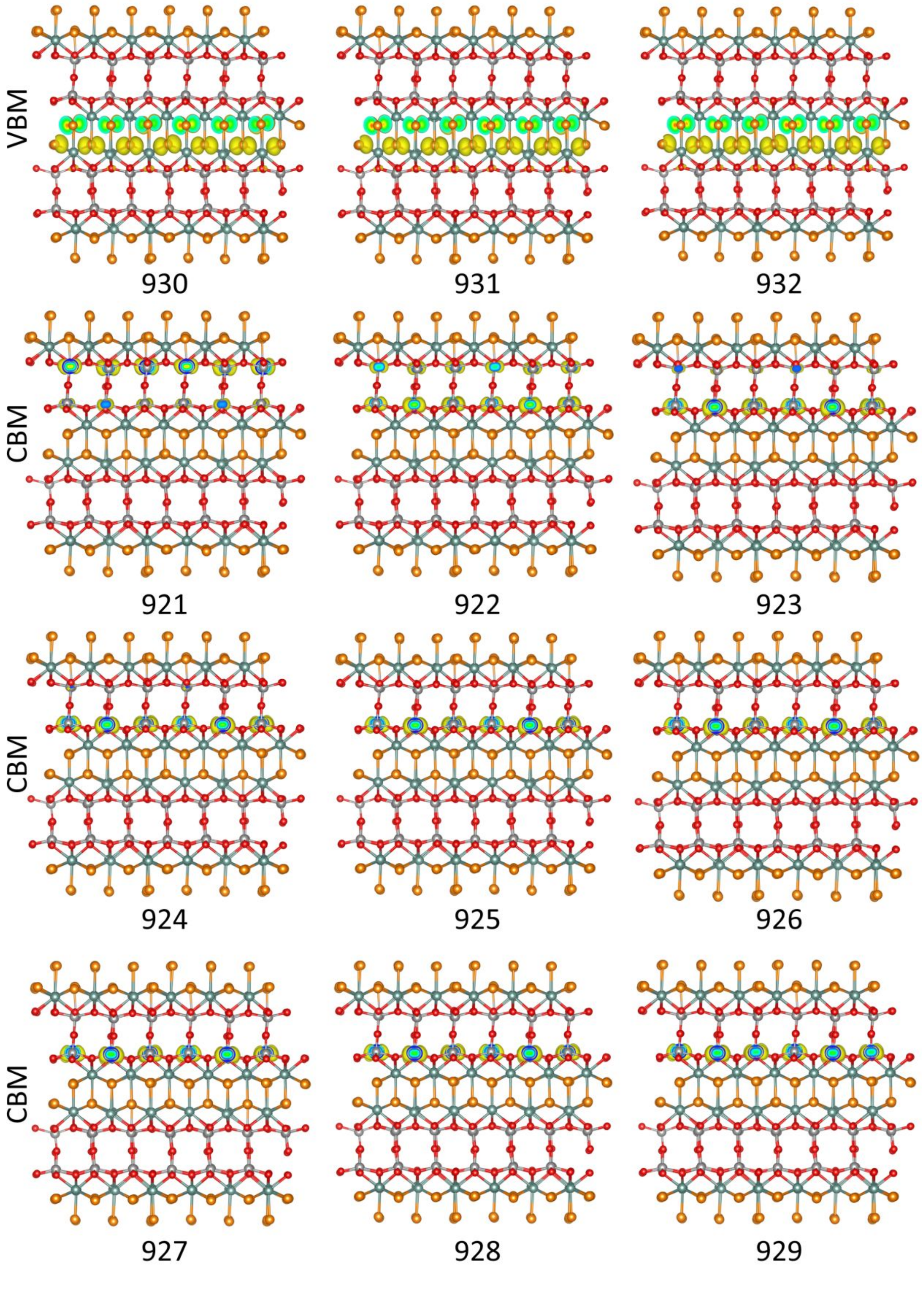
VBM
930
931
932
CBM
921
922
923
CBM
924
925
926
CBM
927
928
929

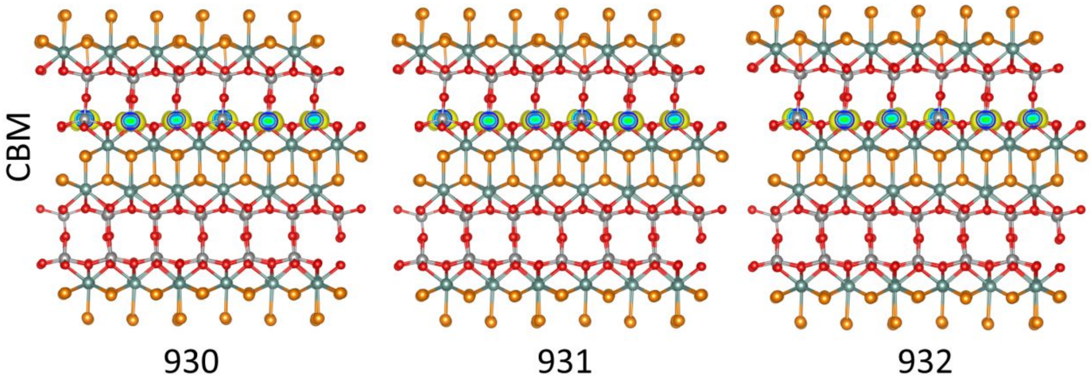

Figure 2S. (a) The NAC's variations over time in NAMD process for $Y_2Ti_2O_5S_2$. (b) The zoom-in view of the highest peak in (a). (c) VBM and CBM states' spatial distribution changing with steps shown in **b**. The value under each snapshot represents the MD step incalculation. We can find that the Band-edge distributions remains relatively stable and temporal asynchrony is evident in the changes of CBM and VBM over time, which is coincide with its low NAC.

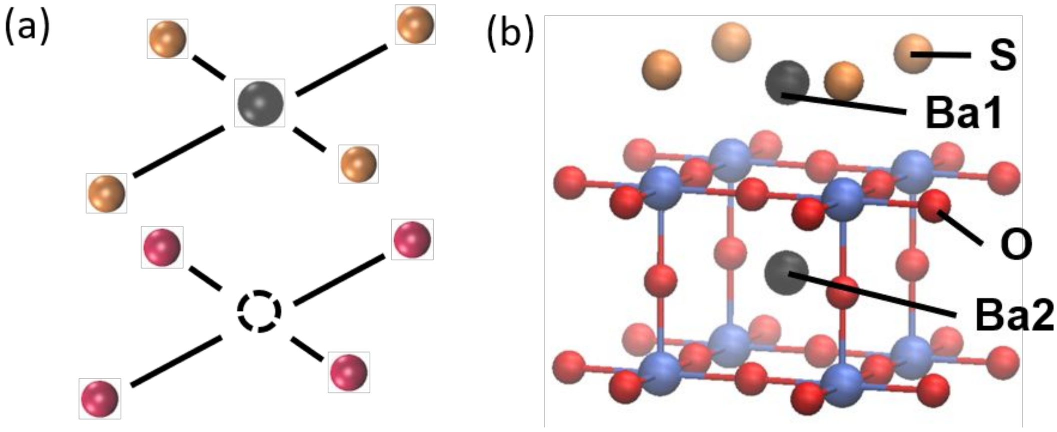

Figure 3S. (a) The local coordination environment around Y atom and cation deficient position in $Y_2Zr_2O_5S_2$. Y atom is coordinated with S atoms and cation-deficient position is coordinated with O atoms. (b) In $Ba_3Zr_2O_5S_2$, Ba atoms' d_{xy} orbital energy-level mainly influenced by the anions (O or S atoms). The radius of S atom is bigger than that of O atom and the interaction between Ba1 and S atoms raised, which will also raise the energy-level of Ba atoms' d_{xy} orbital. Hence the CBM states mainly distribute around Ba2 atoms.

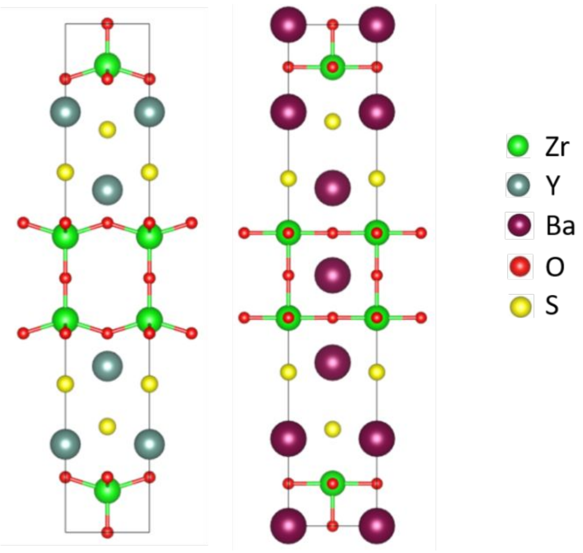

Figure 4S. The side view of $Y_2Zr_2O_5S_2$ (left) and $Ba_3Zr_2O_5S_2$'s (right) crystal structure. We can find that the cation deficient position is filled with Ba atoms in $Ba_3Zr_2O_5S_2$. They both have the similar structure with $Y_2Ti_2O_5S_2$ as shown in Figure 1S.

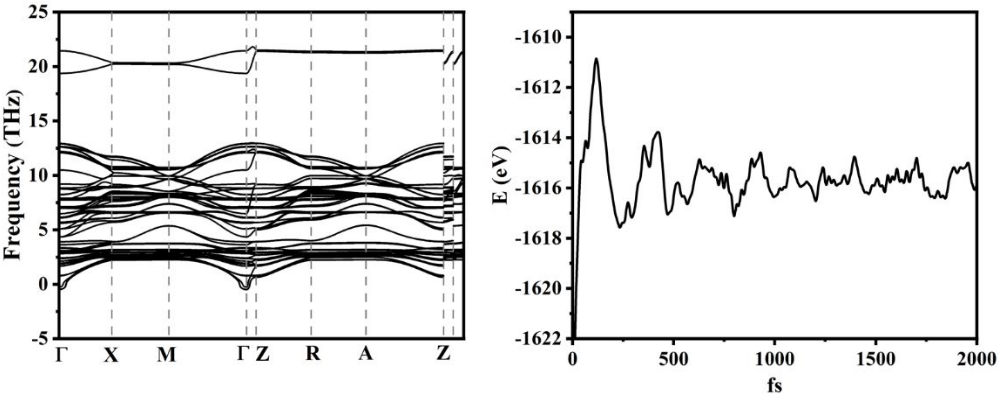

Figure 5S. The phonon spectrum (left) and the energy change at 300K (right) of $Ba_3Zr_2O_5S_2$. The imaginary frequency in phonon spectrum is the systematical error in calculation. We can find that the structure is stable in room temperature.

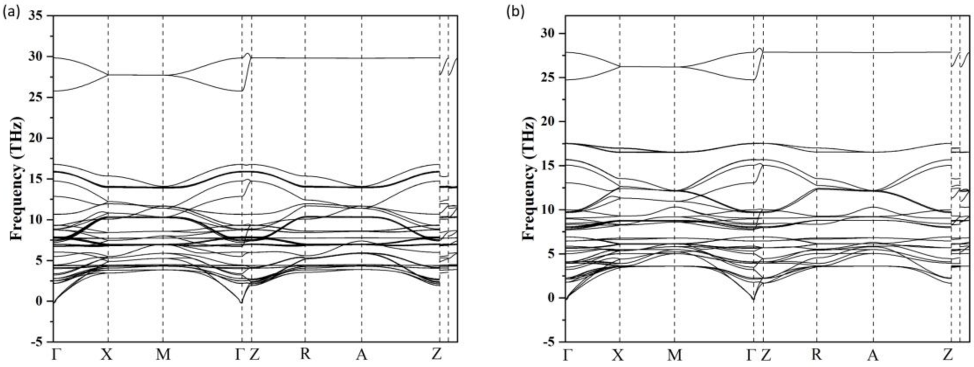

Figure 6S. The phonon spectrums of $Y_2Ti_2O_5S_2$ (left) and $Y_2Zr_2O_5S_2$ (right). Compared with Figure 12S, we can find that their atom vibrations are also similar.

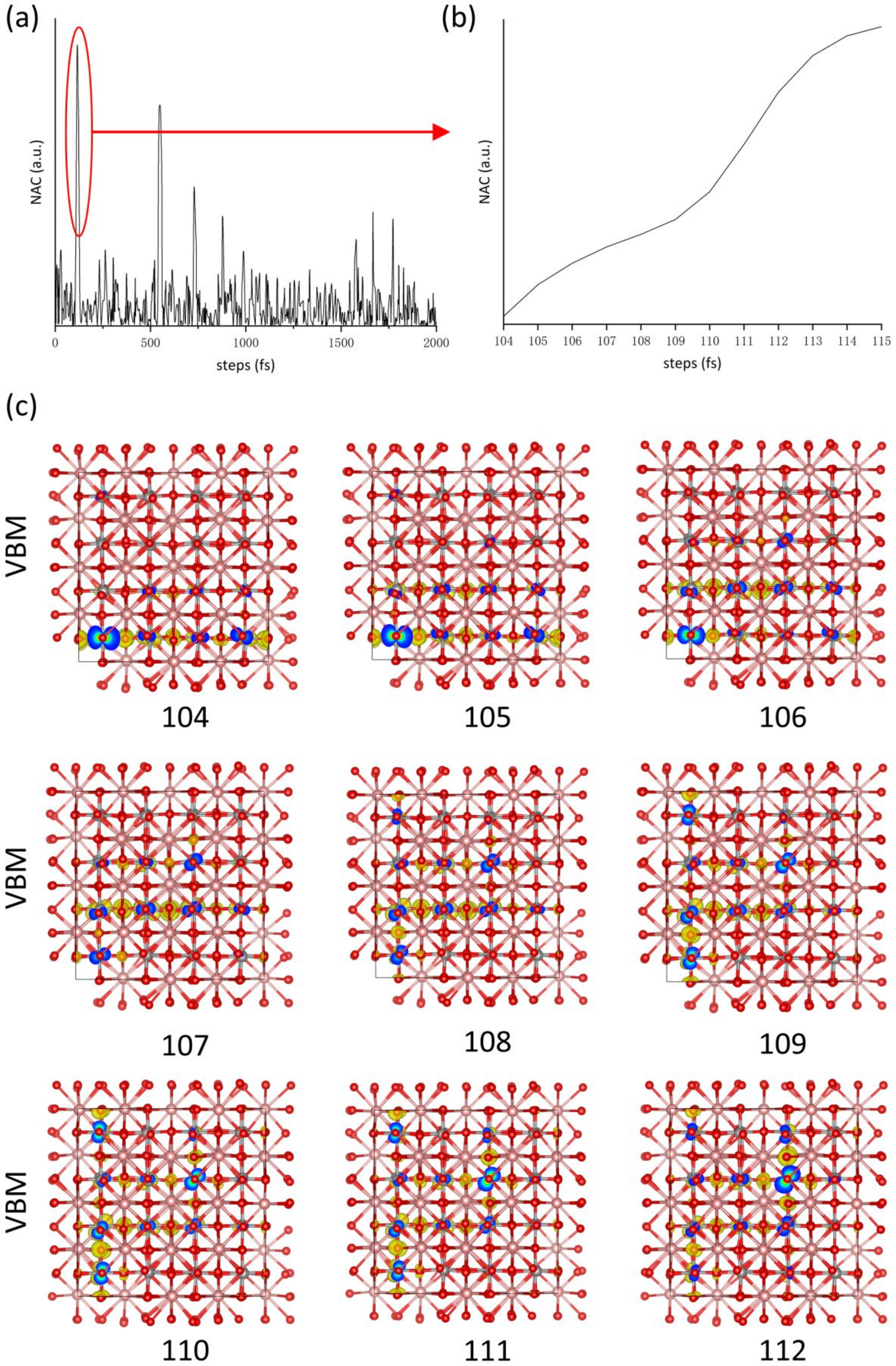
(a)
NAC (a.u.)
steps (fs)
0
500
1000
1500
2000
(b)
NAC (a.u.)
steps (fs)
104
105
106
107
108
109
110
111
112
113
114
115
(c)
VBM
104
105
106
VBM
107
108
109
VBM
110
111
112

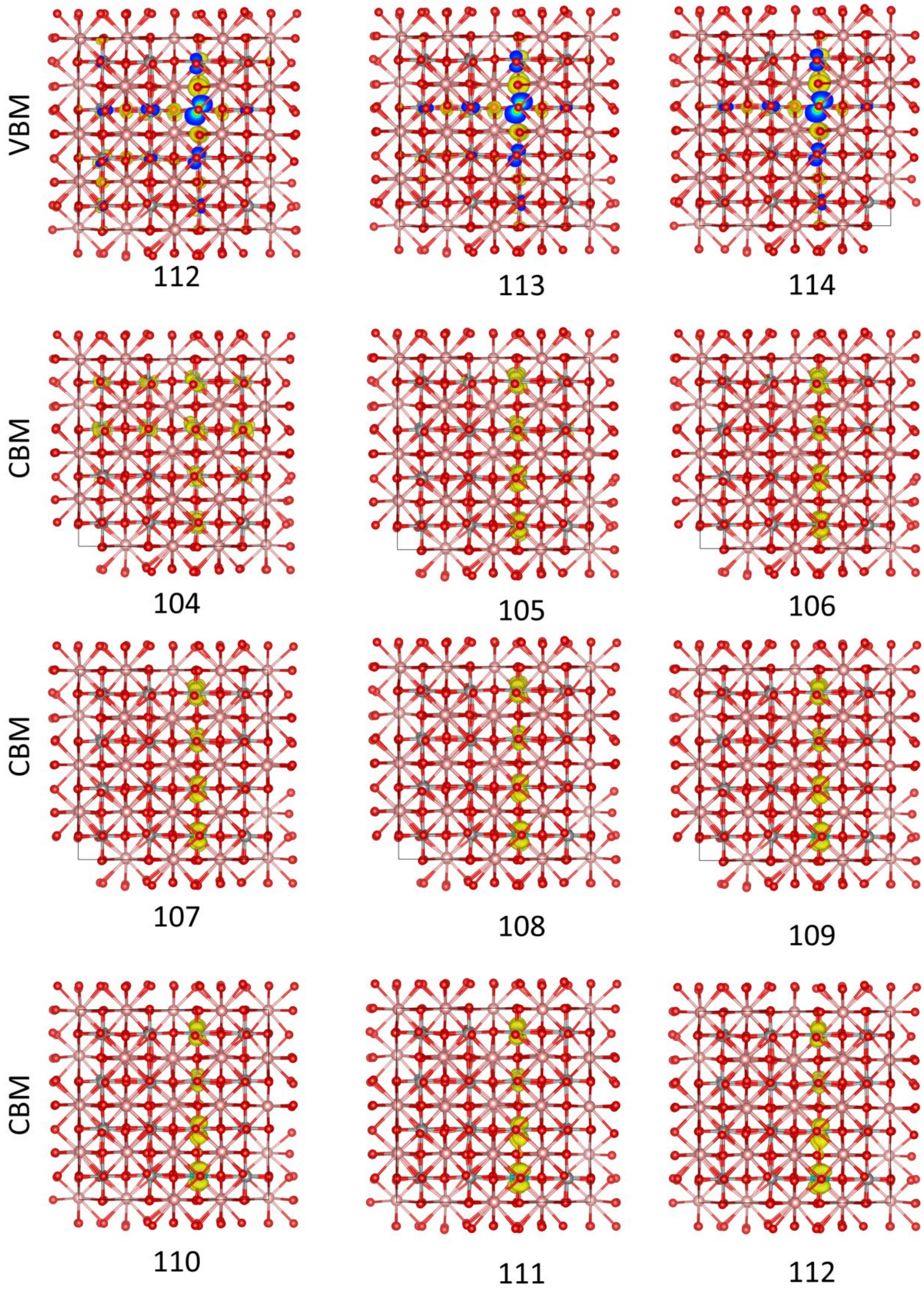
VBM
112
113
114
CBM
104
105
106
CBM
107
108
109
CBM
110
111
112

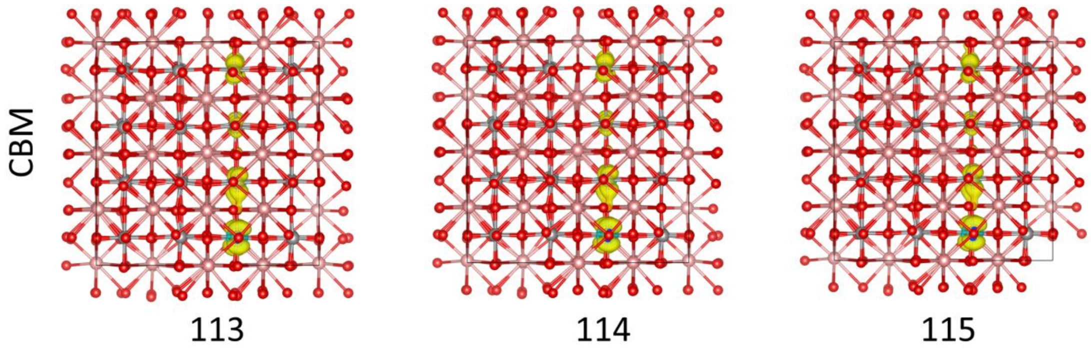

Figure 7S. (a) The NAC's variations over time in NAMD process for $SrTiO_3$. (b) The zoom-in view of the highest peak in (a). (c) VBM and CBM states' spatial distribution changing with steps shown in **b**. The value under each snapshot represents the MD step in calculation. We can find that the changes in VBM states maintain high temporal synchronicity with atomic vibration and ultimately change to the same distribution position as CBM states, which is coincide with $SrTiO_3$'s high NAC.

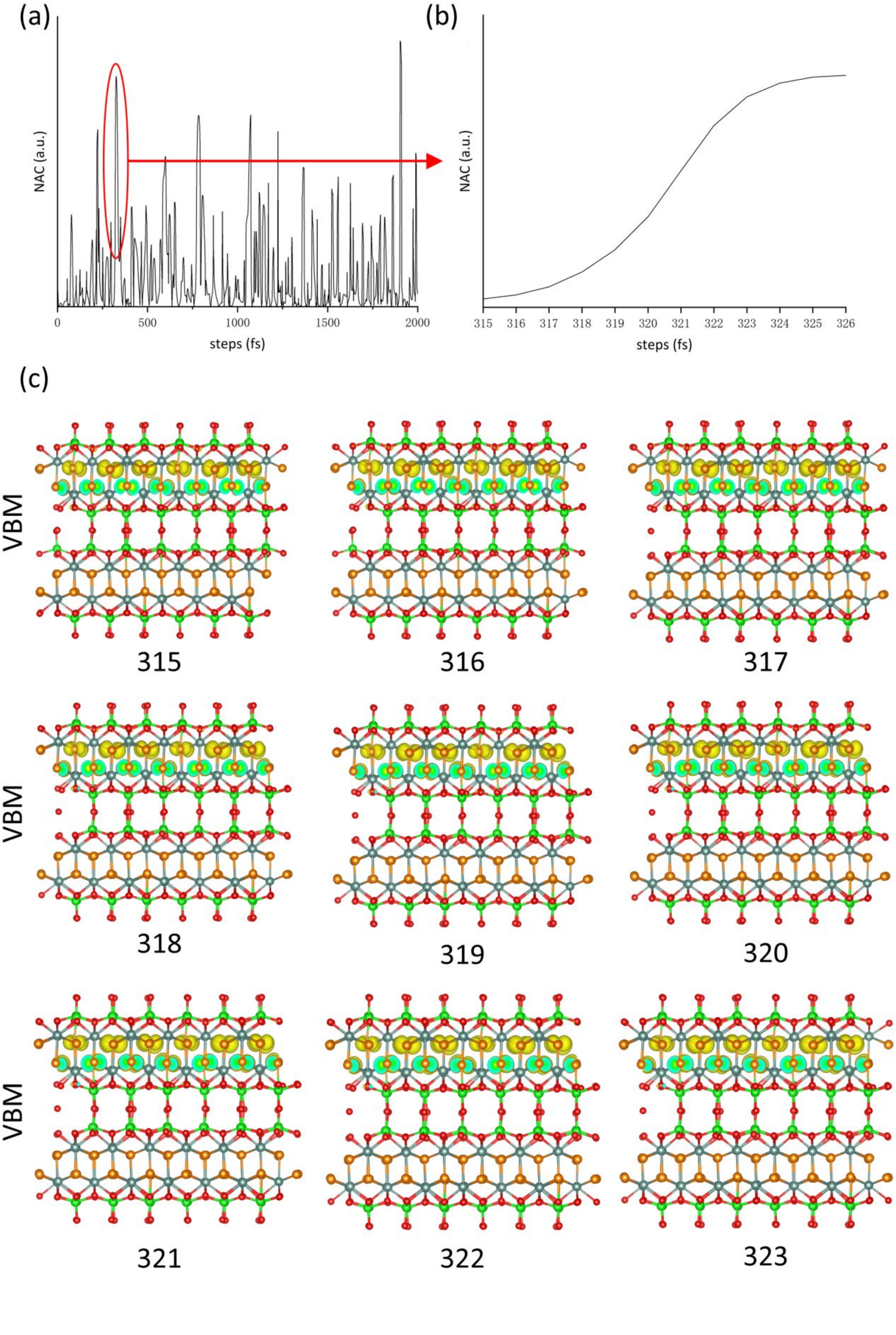
(a)
NAC (a.u.)
steps (fs)
0
500
1000
1500
2000
(b)
NAC (a.u.)
steps (fs)
315
316
317
318
319
320
321
322
323
324
325
326
(c)
VBM
315
316
317
VBM
318
319
320
VBM
321
322
323

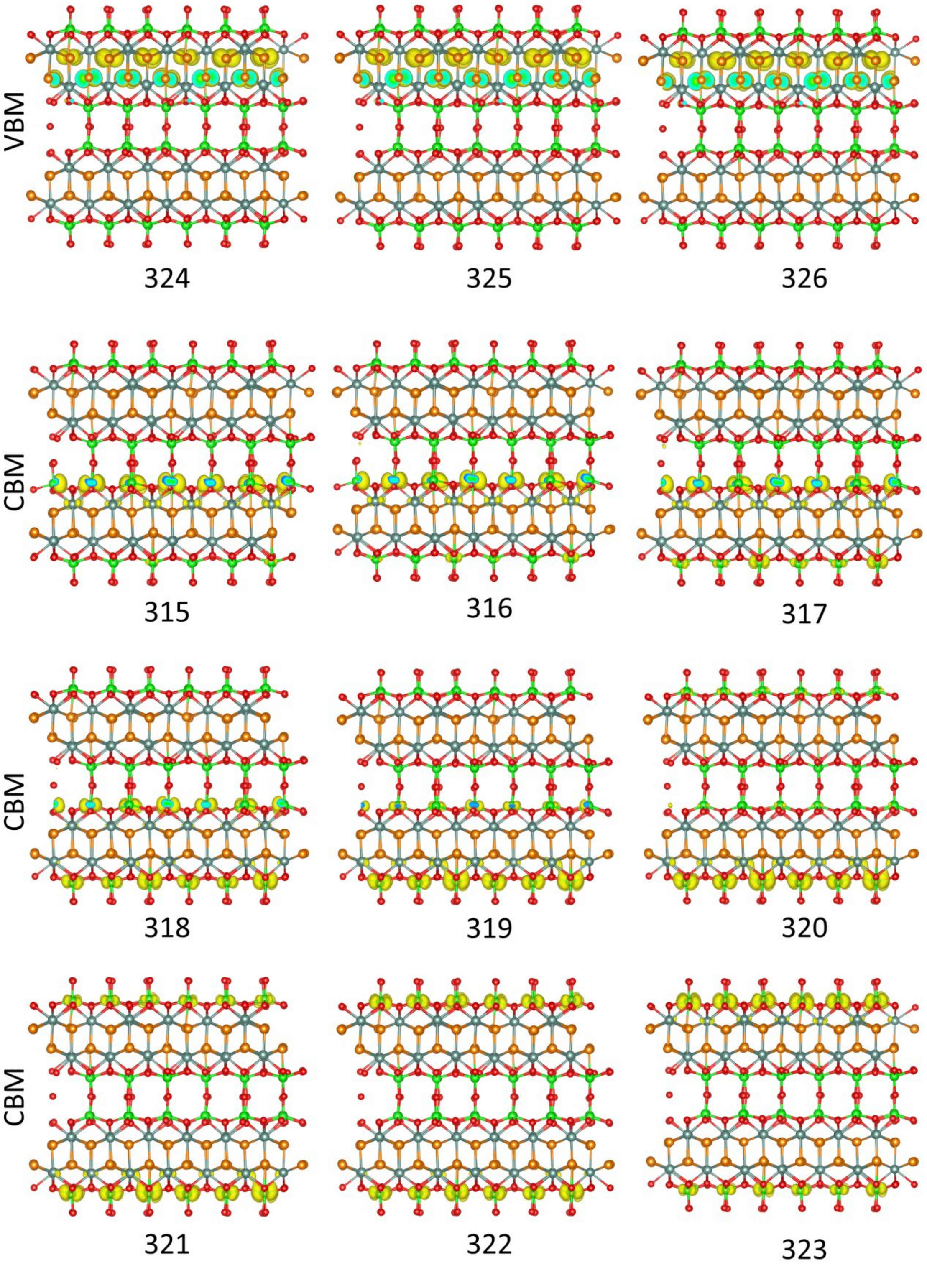
VBM
324
325
326
CBM
315
316
317
CBM
318
319
320
CBM
321
322
323

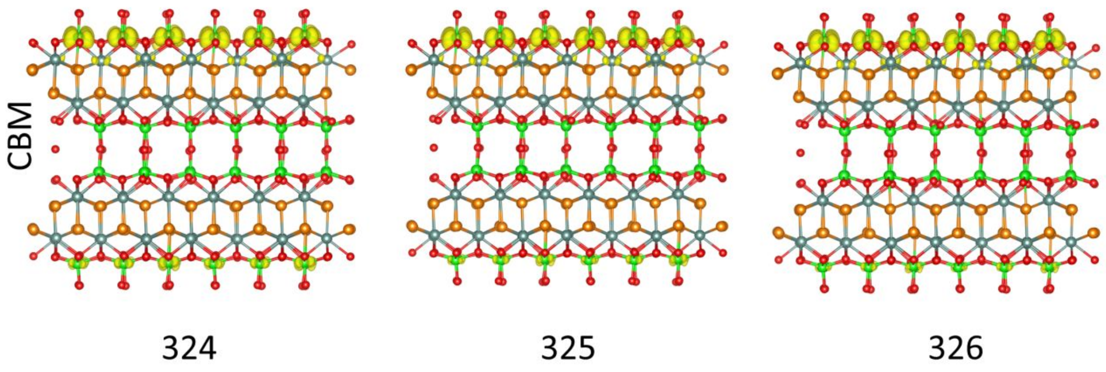

Figure 8S. (a) The NAC's variations over time in NAMD process for $Y_2Zr_2O_5S_2$. (b) The zoom-in view of the highest peak in (a). (c) VBM and CBM states' spatial distribution changing with steps shown in **b**. The value under each snapshot represents the MD step in calculation. We can find that there is a partial distribution of CBM states on Y atoms, which indicate the higher NAC than $Y_2Ti_2O_5S_2$. Besides, the Band-edge distributions remains relatively stable and temporal asynchrony is still evident in the changes of CBM and VBM over time.

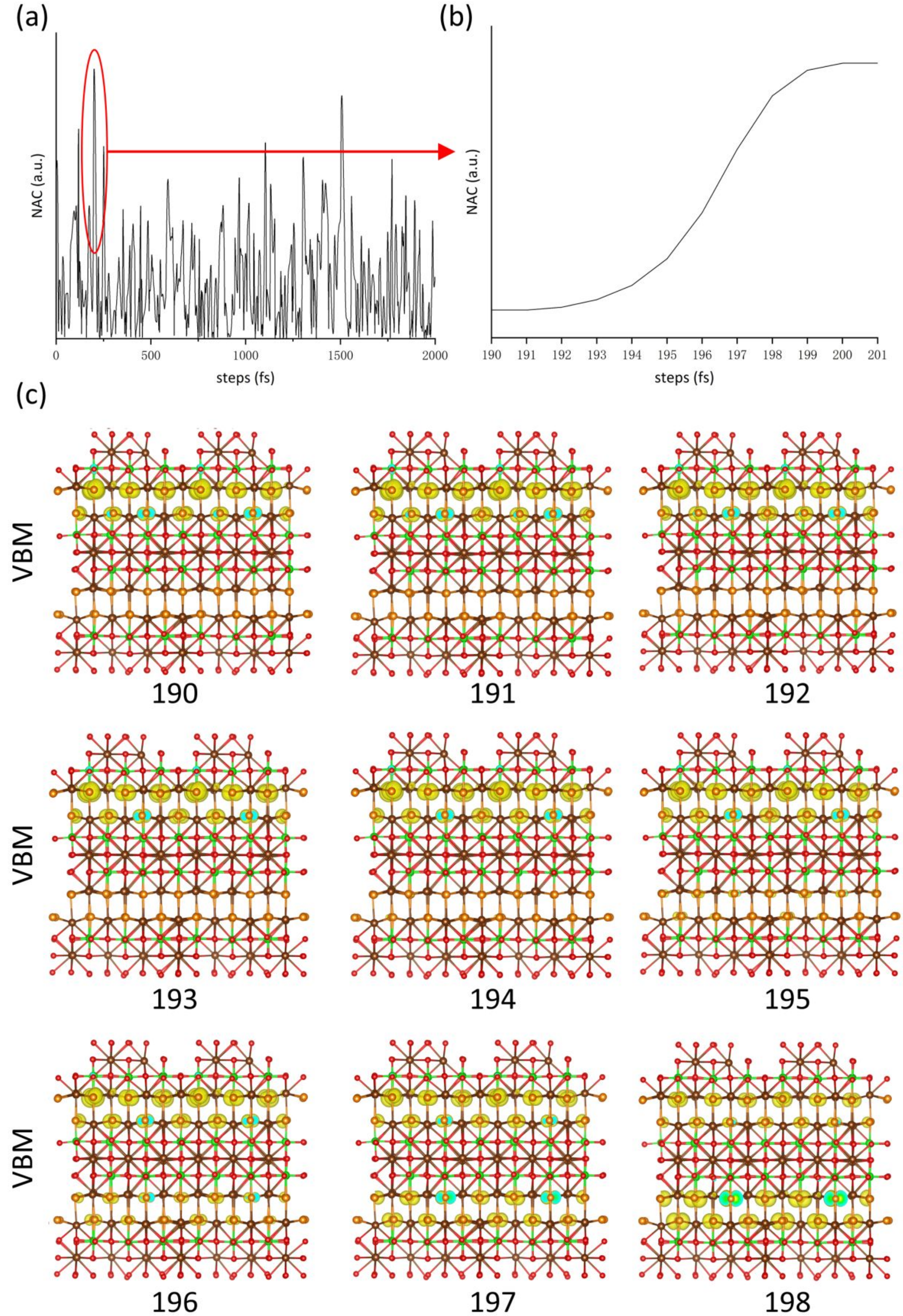
(a)
NAC (a.u.)
0
500
1000
1500
2000
steps (fs)
(b)
NAC (a.u.)
190
191
192
193
194
195
196
197
198
199
200
201
steps (fs)
(c)
VBM
190
191
192
VBM
193
194
195
VBM
196
197
198

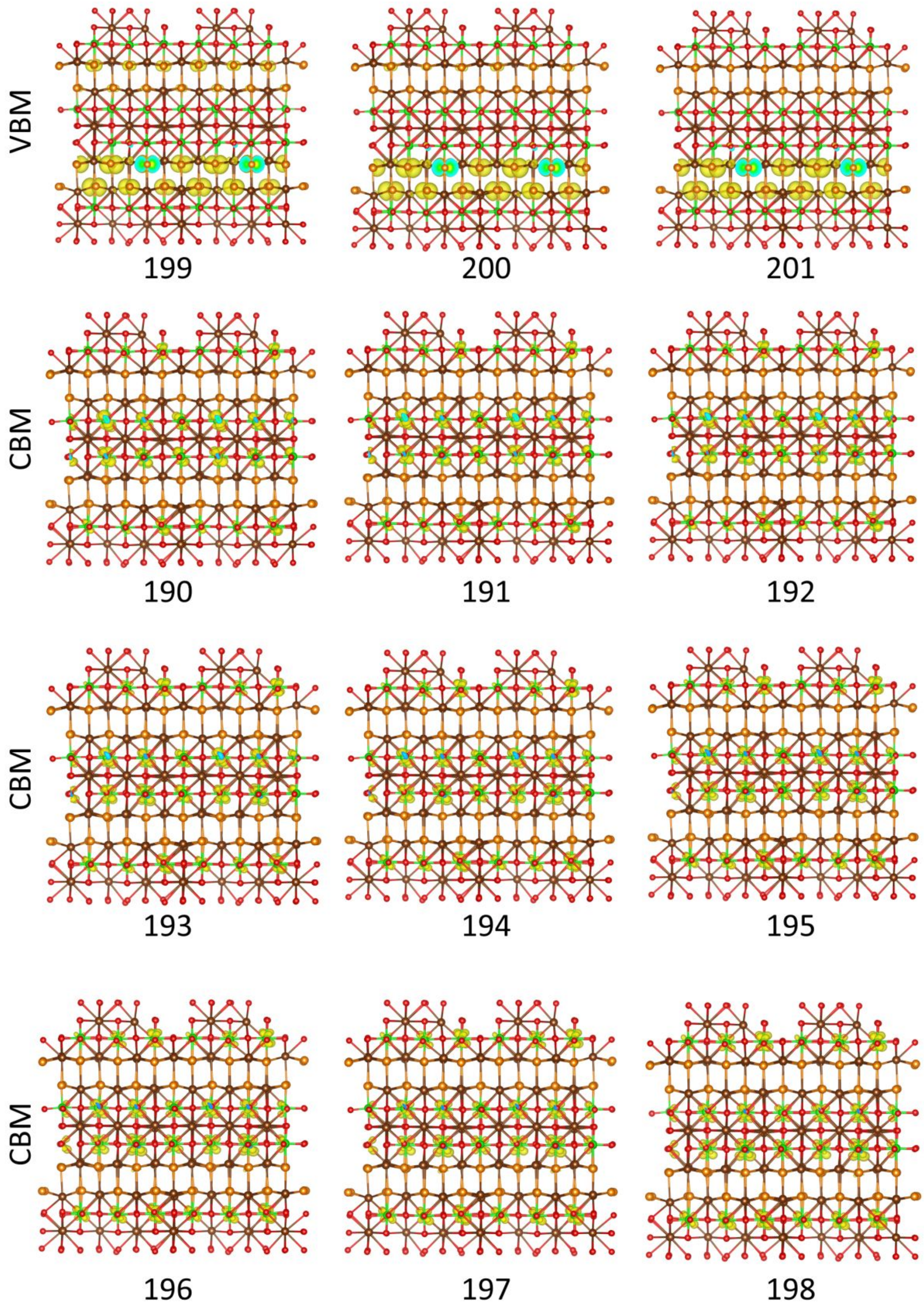
VBM
199
200
201
CBM
190
191
192
CBM
193
194
195
CBM
196
197
198

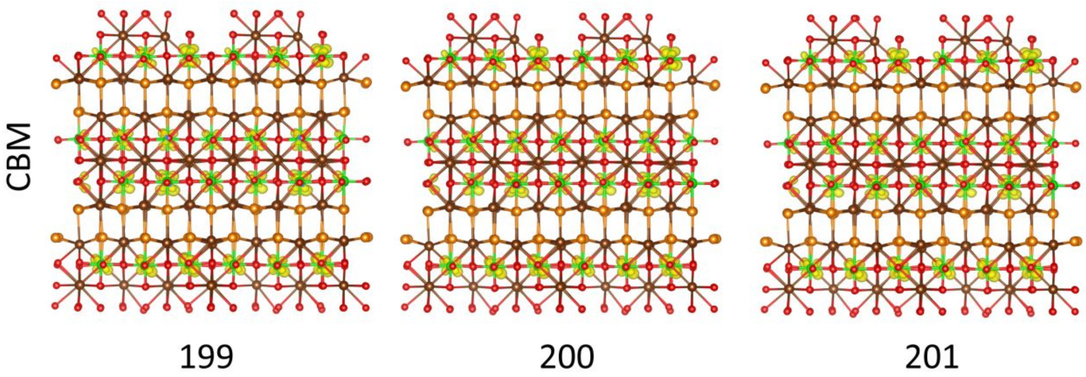

Figure 9S. (a) The NAC's variations over time in NAMD process for $Ba_3Zr_2O_5S_2$. (b) The zoom-in view of the highest peak in (a). (c) VBM and CBM states' spatial distribution changing with steps shown in **b**. The value under each snapshot represents the MD step in calculation. We can also find that the Band-edge distributions remains relatively stable and temporal asynchrony is evident in the changes of CBM and VBM over time, which is coincide with its ultra-low NAC.

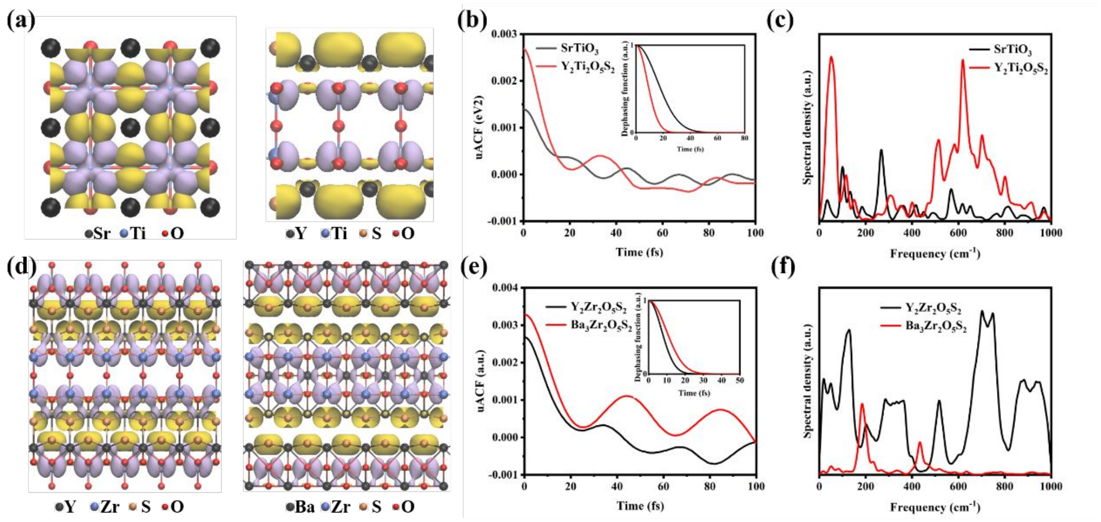

Figure 10S. (a) The overlap between VBM (yellow) and CBM (lavender) in $SrTiO_3$ (left) and $Y_2Ti_2O_5S_2$ (right) with charge densities' isosurface value 0.001 e/Å^3. (d) The overlap between VBM and CBM in $Y_2Zr_2O_5S_2$ (left) and $Ba_3Zr_2O_5S_2$ (right) with isosurface value 0.001 e/Å^3. (b)(e) The un-normalized autocorrelation functions of the energy gap fluctuations at 300K in $SrTiO_3$ (black line) vs Y_2Ti_2O5S2 (red line) (b) and in $Y_2Zr_2O_5S_2$ vs $Ba_3Zr_2O_5S_2$ (e). (c)(f) Influence spectra for $SrTiO_3$ vs $Y_2Ti_2O_5S_2$ (c) and for $Y_2Zr_2O_5S_2$ vs $Ba_3Zr_2O_5S_2$ (f).

In order to study the influences of intrinsic separation on NAC, we have procced the band-gap and NAC data with Mathematical methods in Figure 6S. In the partial charge density of VBM-CBM (Figure 6S), we can find that the CBM have a great overlap with VBM around Ti–O bond in $SrTiO_3$. The overlap in $Y_2Ti_2O_5S_2$ is extremely low with the same isosurface value. **b** presents the unnormalized autocorrelation functions (uACF) of the phonon-induced fluctuations of the energy gaps in $SrTiO_3$ and $Y_2Ti_2O_5S_2$. The Ti–O and Y–S vibration modes in $Y_2Ti_2O_5S_2$ are both responsible for its large gap evoluations and finally NAC variations. The large slope at the beginning of uACF in $Y_2Ti_2O_5S_2$ is attributed to the weak correlation of states at band edges. Pure-dephasing time, τ, is obtained by Gaussian fitting of the pure-dephasing time functions, exp[–0.5(t/τ)], and represents elastic electron-phonon scattering. We can find that the coherence decay fast with the weak correlation of band-edge states, hence the dephasing time in $Y_2Ti_2O_5S_2$ (8.1 fs) is much shorter than that in $SrTiO_3$ (15.4 fs) as collected in Table 1. The Fourier transforms (FTs) of the NAC autocorrelation functions, named as influenced spectral densities, are also plotted (**c**). The data shown in **c** demonstrate that the influence of acoustic branch (low frequence) raised and Ti–O vibration still play a major role in carrier recombination because there is still a few overlap between CBM and VBM around Ti–O bond in $Y_2Ti_2O_5S_2$ (**a** right). We designed $Y_2Zr_2O_5S_2$ to see the influence of the

localization of band-edge states **d** on NAC. The influence spectrum of $Y_2Zr_2O_5S_2$ **f** shows that the influence become significant in full-frequency domain as the overlaps mainly happened between rock-salt layer and perovskite layer **d**. As an optimized choice for band-edge localization, $Ba_3Zr_2O_5S_2$ was designed. Figure d shows that the CBM distribution that used for recombination in rock-salt layer in $Y_2Zr_2O_5S_2$ disappear and they change to distribute around Ba atoms inside perovskite layer in $Ba_3Zr_2O_5S_2$. Figure e shows that the dephasing become slow than $Y_2Zr_2O_5S_2$ as fewer fluctuation **f** can influence the coherence. **f** shows that the phonon modes that participate in recombination of carriers become much few, corresponding to its extremely low NAC (0.18 meV in Table 1) and small NAC fluctuation.

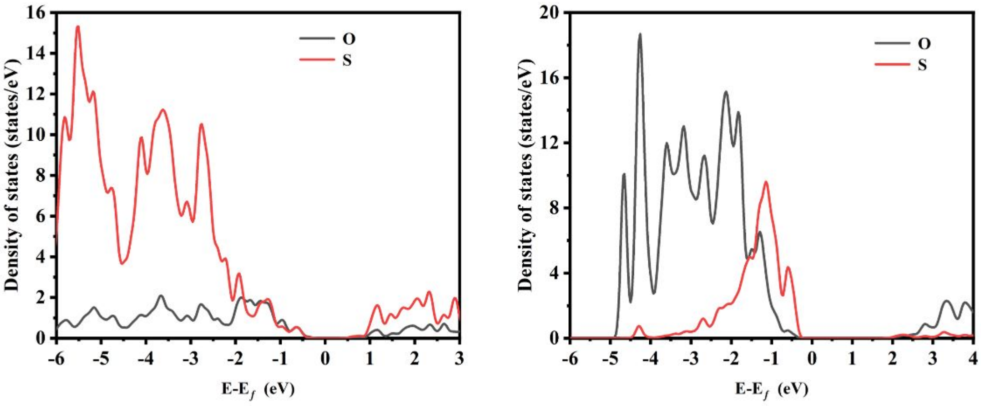

Figure 11S. The projected density of states of $Y_2Ti_2O_5S_2$ (left) and $Ba_3Zr_2O_5S_2$ (right). The Fermi energy is set to zero. Near the Fermi energy, the peak shapes of S and O are similar, indicating the presence of covalent bonds between S and O atoms.

The calculation of hopping probilities between two states

Hefei-named packages are used based on hopping probabilities between the two states obtained as[16]:

$$P_{j\to k}(t,\Delta t) = \frac{2\mathcal{R}\left[c_j^* c_k \boldsymbol{d}_{jk}\right]\Delta t}{c_j^* c_j} \qquad \text{E1}$$

$$\boldsymbol{d}_{jk} = \left\langle \varphi_j \right| \frac{\partial}{\partial t} \left| \varphi_k \right\rangle = \sum_I \frac{\left\langle \varphi_j \middle| \nabla_{\mathbf{R}_I} H \middle| \varphi_k \right\rangle}{\varepsilon_k - \varepsilon_j} \cdot \dot{\mathbf{R}}_I \qquad \text{E2}$$

c are the coefficients of wave functions. $\mathbf{d}_{jk}$ is the NAC between Kohn–Sham states j and k. H is the Kohn-Sham Hamiltonian, φ_j, φ_k, ε_k and ε_j are the wave functions and eigenvalues for electron states j and k. $\dot{\mathbf{R}}_I$ is velocity vector of the nuclei. $\left\langle \varphi_j \middle| \nabla_{\mathbf{R}_I} H \middle| \varphi_k \right\rangle$ expresses the time-dependent orbital coupling including the overlap of two states. $\varepsilon_k - \varepsilon_j$ express the bandgap here. The elastic scattering can be presented by the decay time in pure-dephasing function and the unnormalized autocorrelation functions (uACF). The uACF can be written as:[14, 17]

$$\mathrm{C}(t) = \ < \Delta E(t)\Delta E(0) >_T \qquad \text{E3}$$

where $\Delta E(t) = E(t) -< E >$ is the fluctuation of the energy gap between the two states forming a coherent superposition from its average value. The pure-dephasing function is given by:

$$\mathrm{D}(t) = \exp\left[-\frac{1}{\hbar^2}\int_0^t dt' \int_0^t dt'' C(t'')\right] \qquad \text{E4}$$

Fourier transforms (FTs) of the fluctuations for VBM-CBM energy gaps induced by phonon[13, 17, 18], can reflect the phonon mode that participates in e-ph coupling and is given by:

$$I(\omega) = \frac{1}{2\pi}\left|\int_{-\infty}^{+\infty} dt e^{-i\omega t} C(t)\right|^2 \qquad \text{E5}$$

Supplementary References

1 Sirtl, M. T.; Hooijer, R.; Armer, M.; Ebadi, F. G.; Mohammadi, M.; Maheu, C.; Weis, A.; van Gorkom, B. T.; Haringer, S.; Janssen, R. A. J.; Mayer, T.; Dyakonov, V.; Tress, W.; Bein, T., 2D/3D Hybrid $Cs_2AgBiBr_6$ Double Perovskite Solar Cells: Improved Energy Level Alignment for Higher Contact-Selectivity and Large Open Circuit Voltage. *Adv. Energy Mater.* **2022**, 12, 2103215.

2 Daem, N.; Dewalque, J.; Lang, F.; Maho, A.; Spronck, G.; Henrist, C.; Colson, P.; Stranks, S. D.; Cloots, R., Spray-Coated Lead-Free $Cs_2AgBiBr_6$ Double Perovskite Solar Cells with High Open-Circuit Voltage. *Sol. RRL* **2021**, 5, 2100422.

3 Li, J. B.; Duan, J. L.; Du, J.; Yang, X. Y.; Wang, Y. D.; Yang, P. Z.; Duan, Y. Y.; Tang, Q. W., Alkali Metal Ion-Regulated Lead-free, All-Inorganic Double Perovskites for HTM-free, Carbon-Based Solar Cells. *ACS Appl. Mater. Interfaces* **2020**, 12, 47408–47415.

4 Greul, E.; Petrus, M. L.; Binek, A.; Docampo, P.; Bein, T., Highly Stable, Phase Pure $Cs_2AgBiBr_6$ Double Perovskite Thin Films for Optoelectronic Applications. *J. Mater. Chem. A* **2017**, 5, 19972–19981.

5 Sanchez-Diaz, J.; Sanchez, R. S.; Masi, S.; Krecmarova, M.; Alvarez, A. O.; Barea, E. M.; Rodriguez-Romero, J.; Chirvony, V. S.; Sanchez-Royo, J. F.; Martinez-Pastor, J. P.; Mora-Sero, I., Tin Perovskite Solar Cells with >1,300 h of Operational Stability in N_2 through A Synergistic Chemical Engineering Approach. *Joule* **2022**, 6, 861–883.

6 Jiang, X. Y.; Li, H. S.; Zhou, Q. L.; Wei, Q.; Wei, M. Y.; Jiang, L. Z.; Wang, Z.; Peng, Z. J.; Wang, F.; Zang, Z. H.; Xu, K. M.; Hou, Y.; Teale, S.; Zhou, W. J.; Si, R.; Gao, X. Y.; Sargent, E. H.; Ning, Z. J., One-Step Synthesis of SnI_2 • (DMSO)x Adducts for High-Performance Tin Perovskite Solar Cells. J. Am. Chem. Soc. **2021**, 143, 10970–10976.

7 Jiang, X. Y.; Wang, F.; Wei, Q.; Li, H. S.; Shang, Y. Q.; Zhou, W. J.; Wang, C.; Cheng, P. H.; Chen, Q.; Chen, L. W.; Ning, Z. J., Ultra-High Open-Circuit Voltage of Tin Perovskite Solar Cells via An Electron Transporting Layer Design. *Nat. Commun.* **2020**, 11, 1245.

8 Ran, C. X.; Gao, W. Y.; Li, J. R.; Xi, J.;Li, L.; Dai, J. F.; Yang, Y. G.; Gao, X. Y.; Dong, H.; Jiao, B.; Spanopoulos, I.; Malliakas, C. D.; Hou, X.; Kanatzidis, M. G.; Wu, Z. X., Conjugated Organic Cations Enable Efficient Self-Healing $FASnI_3$ Solar Cells. *Joule* **2019**, 3, 3072–3087.

9 Kopacic, I.; Friesenbichler, B.; Hoefler, S. F.; Kunert, B.; Plank, H.; Rath, T.; Trimmel, G., Enhanced Performance of Germanium Halide Perovskite Solar Cells through Compositional Engineering. *ACS Appl. Energy Mater.* **2018**, 1, 343–347.

10 Elseman, A. M.; Shalan, A. E.; Sajid, S.; Rashad, M. M.; Mostafa'Hassan, A.; Li, M. C., Copper-Substituted Lead Perovskite Materials Constructed with Different Halides for Working $(CH_3NH_3)_2CuX_4$-Based Perovskite Solar Cells from Experimental and Theoretical View. *ACS Appl. Mater. Interfaces* **2018**, 10, 11699–11707.

11 Umar, F.; Zhang, J.; Jin, Z. X.; Muhammad, I.; Yang, X. K.; Deng, H.; Jahangeer, K.; Hu, Q. S.; Song, H. S.; Tang, J., Dimensionality Controlling of $Cs_3Sb_2I_9$ for Efficient

All-Inorganic Planar Thin Film Solar Cells by HCl-Assisted Solution Method. *Adv. Opt. Mater.* **2019**, 7, 1801368.

12 Zhang, Q. H.; Wu, C. C.; Qi, X.; Lv, F.; Zhang, Z. H.; Liu, Y.; Wang, S. F.; Qu, B.; Chen, Z. J.; Xiao, L., Photovoltage Approaching 0.9 V for Planar Heterojunction Silver Bismuth Iodide Solar Cells with Li-TFSI Additive. *ACS Appl. Energy Mater.* **2019**, 2, 3651–3656.

13 Zheng, Q. J.; Chu, W. B.; Zhao, C. Y.; Zhang, L. L.; Guo, H. L.; Wang, Y. N.; Jiang, X.; Zhao, J., Ab initio nonadiabatic molecular dynamics investigations on the excited carriers in condensed matter systems. *WIREs Comput. Mol. Sci.* **2019**, 9, 27.

14 Li, W.; Liu, J.; Bai, F.-Q.; Zhang, H.-X.; Prezhdo, O. V., Hole Trapping by Iodine Interstitial Defects Decreases Free Carrier Losses in Perovskite Solar Cells: A Time-Domain Ab Initio Study. *ACS Energy Lett*. **2017**, 2, 1270–1278.

15 Zhang, Z.; Fang, W. H.; Tokina, M. V.; Long, R.; Prezhdo, O. V., Rapid Decoherence Suppresses Charge Recombination in Multi-Layer 2D Halide Perovskites: Time-Domain Ab Initio Analysis. *Nano Lett.* **2018**, 18, 2459–2466.

16 Li, W.; Tang, J.; Casanova, D.; Prezhdo, O. V., Time-Domain ab Initio Analysis Rationalizes the Unusual Temperature Dependence of Charge Carrier Relaxation in Lead Halide Perovskite. *ACS Energy Lett.* **2018**, 3, 2713–2720.

17 Long, R.; Fang, W.; Prezhdo, O. V., Moderate Humidity Delays Electron-Hole Recombination in Hybrid Organic-Inorganic Perovskites: Time-Domain Ab Initio Simulations Rationalize Experiments. *J. Phys. Chem. Lett.* **2016**, 7, 3215–3222.
